# Revisiting the critical roles of reactive microglia in traumatic brain injury

**DOI:** 10.1097/JS9.0000000000002420

**Published:** 2025-05-12

**Authors:** Jing-Yu Zhao, Yang Zhou, Chao-Wen Zhou, Ke-Bin Zhan, Ming Yang, Ming Wen, Ling-Qiang Zhu

**Affiliations:** a Department of Neurosurgery, Wuhan Hankou Hospital, Hankou Hospital Affiliated to Wuhan University of Science and Technology, Jiang`an District, Wuhan, People’s Republic of China; b Department of Pathophysiology, Tongji Medical College, Huazhong University of Science and Technology, Wuhan, People’s Republic of China; c Department of Neurology, The Second Affiliated Hospital, Hengyang Medical School, University of South China, Hengyang, People’s Republic of China.

**Keywords:** central nervous system, microglia, traumatic brain injury

## Abstract

Traumatic brain injury (TBI) triggers a complex neuroinflammatory cascade, with microglia serving as key regulators of both pathological damage and tissue structural restoration. Despite extensive research, the precise temporal evolution of microglial activation and its implications for long-term neurological outcomes remain incompletely understood. Here, we provide a comprehensive review of the molecular and cellular mechanisms underlying microglial responses in TBI, highlighting their role in neuroinflammation, neurogenesis, and tissue remodeling. We systematically compare clinical and preclinical TBI classifications, lesion patterns, and animal modeling strategies, evaluating their translational relevance. Furthermore, we explore the limitations of the conventional M1/M2 dichotomy and emphasize recent insights from single-cell transcriptomic analyses that reveal distinct microglial subpopulations across different injury phases. Finally, we discuss current therapeutic strategies targeting microglial modulation and propose future directions for neuroimmune interventions in TBI. By integrating findings from experimental and clinical studies, this review aims to bridge mechanistic insights with therapeutic advancements, paving the way for precision-targeted neuroimmune therapies.

**HIGHLIGHTS**

- Microglial responses in traumatic brain injury (TBI): This review explores the molecular and cellular mechanisms of microglial responses in TBI, emphasizing their crucial roles in neuroinflammation, neurogenesis, and tissue remodeling.
- Insights from single-cell transcriptomics: It highlights recent findings from single-cell transcriptomic analyses, revealing distinct microglial subpopulations across different injury phases, which challenges the conventional M1/M2 dichotomy.
- Therapeutic strategies and future directions: The review discusses current therapeutic strategies targeting microglial modulation and proposes future research directions for neuroimmune interventions in TBI, aiming to advance precision-targeted neuroimmune therapies.

## Introduction

Traumatic brain injury (TBI) remains a major public health concern, leading to long-term neurological impairments and an increased risk of neurodegenerative diseases. As the primary innate immune cells of the central nervous system (CNS), microglia play a pivotal role in orchestrating the neuroinflammatory response to TBI. Upon injury, microglia rapidly activate and undergo dynamic phenotypic changes that can either exacerbate secondary damage or promote neuroprotection and tissue structural restoration. Elucidating the precise molecular and cellular mechanisms underlying microglial activation is essential for developing effective therapeutic strategies.

Despite extensive research, several challenges hinder the clinical translation of experimental findings. A key limitation is the reliance on the conventional M1/M2 polarization framework, which oversimplifies microglial heterogeneity and fails to capture the full spectrum of activation states across different injury phases. Recent advances in single-cell transcriptomics have provided unprecedented insights into the temporal and injury-severity-dependent diversity of microglial responses, revealing distinct subpopulations that may serve as therapeutic targets. In our study, we performed single-cell RNA sequencing analysis to characterize TBI-associated microglial states and identified a unique subset of microglia, termed trauma-associated microglia (TAM). Importantly, TAMs exhibit a phase-dependent functional shift, suggesting their potential as dynamic therapeutic targets.

However, the clinical significance of these findings remains to be fully established, necessitating an integrative approach that bridges experimental models with human pathology. This review aims to provide a comprehensive analysis of microglial dynamics in TBI, focusing on their dual role in neuroinflammation and tissue structural restoration. We systematically compare preclinical and clinical TBI models, assess their translational relevance, and highlight the limitations of existing paradigms. By integrating current knowledge from experimental and clinical studies, we propose a temporally and model-specific framework for microglial modulation, with the goal of optimizing neuroimmune responses and improving long-term outcomes in TBI patients.

## Literature retrieval strategy

The articles used in this review were retrieved mainly using keywords such as “traumatic brain injury (TBI),” microglia”, animal models”, “neuroinflammation,” “signalling pathways,” and “central nervous system,” “intervention” with Boolean operators (AND/OR) applied to identify intersections (e.g., “TBI AND microglia AND neuroinflammation”). Searches were conducted across PubMed, prioritizing studies published between 2015 and 2025 to reflect post-scRNA-seq methodological advancements. To enhance specificity, supplementary terms were incorporated, including microglial polarization”, “damage-associated molecular patterns (DAMPs),” “cytokines,” “single-cell RNA sequencing (scRNA-seq),” “crosstalk,” “angiogenesis,” “neurogenesis,” “inflammasome” and “neuroinflammatory regulation.” Non-peer-reviewed articles, non-English publications, and reviews were systematically excluded to maintain focus on primary mechanistic evidence.

## TBI

TBI arises following the application of external mechanical forces to the craniofacial or cervical regions^[^1^]^. Global TBI incidence approximates 69 million annual cases, imposing substantial socioeconomic burdens on healthcare systems^[^2^]^. TBI manifests through distinct biomechanical injury patterns, with notable clinical heterogeneity stemming from divergent etiologies, pathological mechanisms and recovery trajectories^[^3-5^]^. Within clinical practice, the Glasgow Coma Scale (GCS)^[^6,7^]^ and its extended iteration, the Glasgow Coma Scale Extended^[^6^]^ are employed to stratify TBI severity and predict clinical trajectories. The GCS remains the predominant clinical stratification tool for TBI, categorizing injuries into mild (14–15), moderate (9–13), and severe (3–8) based on cumulative scoring^[^8,9^]^. This tripartite system integrates ocular responsiveness, verbal performance, and motor reactions to derive a composite score. Nevertheless, diagnostic uncertainties persist, particularly in differentiating mild versus moderate TBI categories. A notable example involves inconsistent clinical interpretations of threshold scores, exemplified by ongoing debates regarding the classification of GCS 13 cases^[^10^]^. Furthermore, this approach fails to account for individual variations in pathoanatomical characteristics or pathophysiological mechanisms, while being compromised by pharmacological interference, substance intoxication, and airway management interventions such as tracheal intubation^[^11,12^]^. The Injury Severity Score (ISS) and New Injury Severity Score (NISS) are globally recognized trauma stratification tools, with the Maximum Abbreviated Injury Scale (MAIS) offering enhanced accuracy for head injury assessment^[^13-15^]^. ISS and NISS categorize the body into anatomical regions, assigning each a severity grade from 1 (minor) to 6 (unsurvivable) based on lesion characteristics^[^13,14^]^. These scores predict post-traumatic mortality, morbidity, and hospitalization duration. MAIS, a subcomponent of ISS, quantifies injury severity within a single anatomical region. A MAIS score of 5 indicates critical tissue damage and correlates strongly with adverse clinical outcomes^[^15^]^. Contemporary TBI research prioritizes enhanced phenotyping methodologies through systematic integration of supplementary clinical parameters with the GCS, complemented by cutting-edge neuroimaging protocols, multimodal physiological surveillance, hematological biomarker profiling, and genomic characterization^[^16^]^. Neuroimaging techniques are critical for delineating neuropathological features and diagnosing TBI. Computed tomography (CT) scans are routinely employed in emergency settings post-injury to assess acute cerebral damage. To enhance prognostic accuracy, standardized grading systems integrating neuroimaging data have been established. The Marshall Scale, a widely adopted classification framework, stratifies TBI severity into six distinct categories based on CT-derived morphological abnormalities^[^17^]^. Mild traumatic brain injury (mTBI) may involve transient confusion and memory impairment peri-injury, accompanied by loss of consciousness (<30 minutes) and post-30-minute GCS scores of 13–15, with CT or magnetic resonance imaging (MRI) potentially revealing no structural abnormalities^[^1,18^]^. Notably, elevated serum biomarker levels in individuals with normal CT findings may indicate structural brain damage, corroborated by MRI abnormalities observed in up to 30% of mild TBI cases^[^16^]^. The Glasgow Outcome Scale (GOS) served as the principal clinical endpoint, employing a 5-tiered scoring system to quantify functional impairment following neurological trauma^[^19^]^. Among 237 complicated mild TBI cases assessed at 15-month follow-up, 63% demonstrated favorable GOS-defined recovery, with disability persisting in 33% (30% moderate, 3% severe) and mortality occurring in 4.2%^[^20^]^. Mild-to-moderate TBIs constitute nearly 90% of global cases^[^2^]^. Heightened public understanding of mTBI epidemiology in recent years stems largely from documented evidence of significant complication rates among athletic and military veteran populations^[^21^]^. This synthesis focuses on mild-to-moderate TBI pathophysiology, where clinical heterogeneity and diagnostic uncertainties remain unresolved, necessitating enhanced mechanistic stratification to inform targeted therapeutic strategies^[^2^]^. However, experimental TBI research lacks standardized criteria for injury severity assessment, hampering translational applicability of preclinical findings^[^22^]^. Parameters requiring consensus-based standardization encompass: mechanical indices (e.g., peak pressure magnitude and duration)^[^23^]^; neurological alterations (e.g., apnea duration, righting reflex latency, and pinna reflex impairment)^[^24^]^; physiological deviations (e.g., body weight loss and intracranial pressure elevation)^[^25^]^; histopathological markers (e.g., infarct volume quantification and neuronal density reduction); and behavioral deficits assessed via neurological severity scoring or motor function paradigms^[^26^]^. Despite the lack of consensus on standardized injury severity grading, a review consolidates contemporary evidence regarding severity criteria across TBI models, encompassing focal, diffuse, and mixed injury mechanisms. This work further examines the potential application of mortality rates and cerebral infarct volume quantification as objective benchmarks for classifying mild, moderate, and severe TBI^[^22^]^.

Focal brain injury primarily arises from direct mechanical forces such as blunt trauma (e.g., cranial impacts, vehicular collisions, or physical assaults), resulting in localized tissue destruction. In contrast, diffuse injury stems from acceleration-deceleration mechanisms, exemplified by unconstrained head motion during high-speed collisions or blast-induced wave propagation. Mixed injury paradigms encompass trauma typologies associated with falls or sports-related impacts, combining elements of both focal and diffuse pathophysiology^[^22^]^. Focal brain injury, predominantly involving the frontal and temporal lobes, primarily arises from mechanical compression of cerebral tissue at the impact site due to cranial collision forces. This pathomechanism clinically presents with subdural hematoma, epidural hematoma, and hemorrhagic contusions^[^27,28^]^. Focal TBI compromises blood–brain barrier (BBB) integrity, inducing extracellular fluid accumulation through cellular extravasation^[^29^]^. Concurrently, cerebral perfusion dynamics are disrupted, manifesting as pathological hypo- or hyperperfusion states^[^30^]^. Contrecoup brain injury represents a distinct subcategory of focal TBI characterized by predominant cerebral contusions developing contralateral to the site of blunt cranial trauma^[^31,32^]^. Diffuse brain injury typically arises from sudden acceleration-deceleration forces applied to the head, correlating clinically with consciousness impairments secondary to axonal injury, vascular disruption, and cerebral edema^[^9^]^. The neuroanatomical distribution of axonal shearing or persistent focal lesions in diffuse axonal injury (DAI) critically determines clinical outcomes, with predilection sites encompassing the corona radiata, corpus callosum, internal capsule, brainstem, and thalamic regions^[^33^]^. Fatal DAI cases are defined by three pathognomonic neuropathological features: focal corpus callosal lesions, focal rostral brainstem lesions, and widespread axonal damage^[^34^]^. DAI is pathologically characterized by periodic disruptions of axonal transport mechanisms at lesion sites, manifesting as bead-like swellings termed “axonal varicosities” and terminal bulb-shaped enlargements (“axonal bulbs”) – both hallmark histopathological features of DAI^[^35^]^. The multifocal distribution of axonal injury lesions in DAI – rather than truly diffuse – and their detection in concussion patients underscore the necessity for standardized terminology to describe axonal pathology across both concussion and DAI diagnoses^[^36-42^]^. Initiatives have been prioritized to adopt terminology such as traumatic axonal injury or diffuse traumatic axonal injury, aiming to address the terminological limitations of “diffuse” in DAI and explicitly highlight the traumatic etiology underlying axonal pathology in head injury^[^34,35,43,44^]^. A diagnosis of DAI is clinically confirmed when a patient demonstrates post-traumatic loss of consciousness exceeding 6 h, alongside related neurological manifestations^[^45-47^]^. Notably, a subset of patients exhibit discernible evidence of DAI on diffusion-weighted imaging (DWI) metrics despite unremarkable findings on conventional MRI sequences, including susceptibility-weighted imaging (SWI). Conversely, SWI may reveal diffuse vascular pathology in cases without detectable diffusion abnormalities. These observations underscore the complementary diagnostic insights provided by DWI and SWI in evaluating white matter (WM) integrity following TBI^[^48^]^. The advent of diffusion tensor imaging (DTI) has established a quantitative approach for in vivo assessment of WM microstructure, utilizing fractional anisotropy as a biomarker of axonal integrity^[^49^]^. This modality demonstrates enhanced sensitivity in detecting and quantifying DAI, even in patients with structurally normal conventional neuroimaging findings, as evidenced by multiple clinical studies^[^50-54^]^. While DTI metrics are recognized as sensitive biomarkers for detecting microstructural pathology in TBI^[^49,55^]^, emerging evidence highlights substantial heterogeneity in their interstudy reproducibility and observed responses across experimental and clinical cohorts^[^56-58^]^. Diffusion MRI metrics quantifying DAI demonstrate robust prognostic value in predicting trauma-induced neurodegenerative progression^[^59^]^. TBI animal models are categorized into three primary classifications: focal, diffuse, and mixed injuries. Focal injuries, characterized by localized tissue damage, are replicated through controlled cortical impact, penetrating ballistic-like brain injury, and Feeney/Shohami weight-drop paradigms. Diffuse injuries, involving widespread axonal and vascular pathology, are modeled via shock tube-induced primary blast injury or Marmarou/Maryland weight-drop systems. Mixed injury models, combining focal and diffuse pathophysiological features to mimic clinical concussion syndromes, are optimally represented by lateral fluid percussion injury (FPI) paradigms^[^22^]^. The pathophysiology of TBI exhibits multidimensional complexity, with focal, diffuse, and mixed injury subtypes corresponding to distinct biomechanical and molecular cascades. While multimodal neuroimaging techniques (e.g., DTI, SWI) provide complementary tools for precise diagnosis and injury stratification, challenges persist in standardizing terminology and resolving imaging heterogeneity.

Regardless of injury classification (focal, diffuse, or mixed), TBI pathophysiology is fundamentally defined by biphasic injury cascades encompassing primary mechanical damage and secondary molecular pathomechanisms. Primary injury in TBI encompasses pathologically diverse manifestations, including vascular pathologies (contusions, hematomas, hemorrhages), axonal shearing injuries, and cytotoxic edema^[^60^]^. The secondary phase involves multifaceted molecular cascades evolving over hours to days post-trauma, culminating in neuronal death, neuroinflammation, and progressive neurodegeneration. Key mechanisms include glutamate-mediated excitotoxicity; apoptotic signaling; neuroimmune activation with concomitant gliosis; tau hyperphosphorylation; dendritic atrophy; demyelination; necrotic cell death; autoimmune responses; and mitochondrial dysregulation^[^61^]^. Therapeutic management of TBI prioritizes urgent intervention to mitigate secondary damage, as primary mechanical insults are irreversible. Secondary injury mechanisms evolve over minutes to days post-trauma, driven by neuroinflammatory cascades, cerebrovascular dysregulation, and disruption of neural homeostasis – collectively precipitating neuronal apoptosis and structural degeneration^[^62^]^. No pharmacotherapy has demonstrated definitive clinical efficacy in human TBI. Contemporary strategies focus on optimizing cerebral oxygen delivery through prevention of hypoxemia, hypercapnia, and systemic hypotension, coupled with strict maintenance of cerebral perfusion pressure and euvolemia to minimize secondary pathological progression^[^63,64^]^. The primary injury phase transitions into secondary neuroinflammatory cascades, where neurovascular unit dysregulation and peripheral glial activation drive progressive neurodegeneration through cytokine-mediated oxidative stress pathways^[^65^]^. Deficiencies in neurometabolism can lead to chronic neuroinflammatory responses. These responses may lead to various secondary consequences that can persist for months or even years after the initial injury. Such consequences include oxidative stress, cell death, and persistent disruption of the BBB, all of which collectively contribute to widespread neuroinflammation^[^66^]^. Secondary injury is predominantly influenced by neuroinflammation, characterized by an increase in the concentrations of proinflammatory and anti-inflammatory cytokines and chemokines^[^67^]^. Microglia play a central role in post-TBI neuroinflammation, with reactive astrocytes and infiltrating immune cells further shaping the inflammatory response. Persistent microglial activation may exacerbate long-term neuroinflammation and neurodegeneration^[^68^]^.

**Figure 1.**
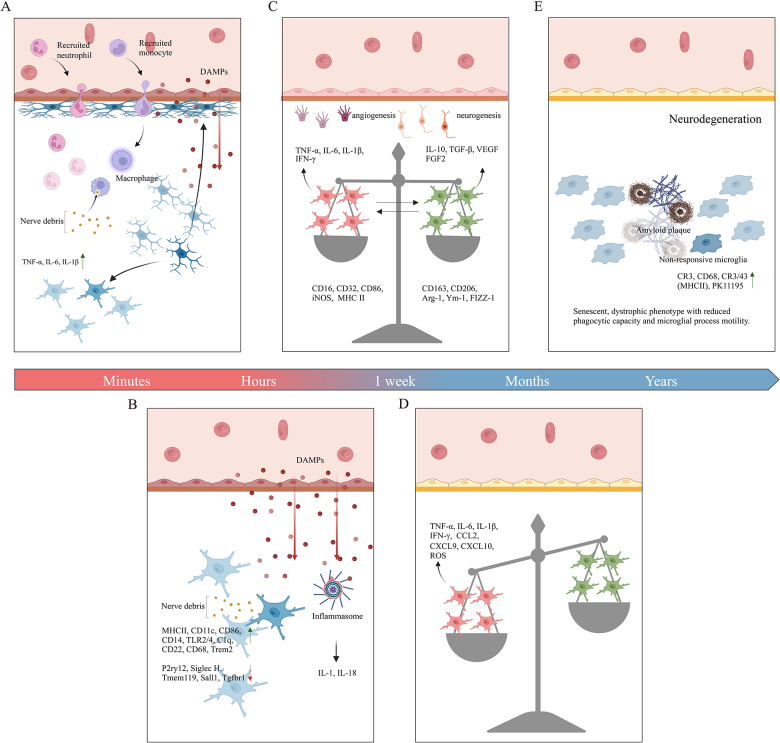
The role of microglia in TBI at different stages. (A) Within the first few minutes after the injury occurs, microglia are crucial as they expand their processes and envelop blood vessels to help restore the integrity of the compromised BBB. The initial phase consists of the rapid activation of resident microglia within the CNS, resulting in the release of pro-inflammatory cytokines and the attraction of peripheral immune cells, including neutrophils and monocytes, to the affected site. (B) In a reactive state, microglia are unable to perform the barrier closure function. Microglia synthesize neuroprotective factors, clear cellular debris, and orchestrate nerve repair processes that contribute to neurological recovery following TBI. (C) The microglia population exhibits a balanced distribution between pro-inflammatory and anti-inflammatory states during the first week. This dynamic process facilitates neurogenesis and enhances the immune response to injury. M2-polarized microglia and macrophages play a potential role in post-traumatic angiogenesis and wound healing. (D) This early response of microglia limits lesion expansion but also produces inflammatory cytokines and ROS, which may adversely affect recovery and contribute to secondary injury if not adequately resolved. (E) TBI accelerates the onset of AD at a younger age, typically several years after the initial injury, and the severity of the injury is associated with an increased risk of developing AD. The activation and functional changes of microglia are considered key mediators in the chronic neurodegenerative pathological evolution following TBI (Image created with BioRender.Com).

TBI accelerates the onset of Alzheimer’s disease (AD) at younger ages, and the severity of the injury is associated with an increased risk of developing AD^[^69^]^. Longitudinal analysis of World War II veterans revealed a dose-dependent association between TBI severity and late-onset AD risk. Individuals exposed to moderate TBI during young adulthood demonstrated 2.3 elevated AD incidence, respectively, after a 40-year latency period compared to non-TBI counterparts, based on validated diagnostic protocols^[^70^]^. The accumulation of amyloid precursor protein (APP) in the axons of TBI survivors, along with diminished phagocytic (M2) capacity and impaired microglial clearance of amyloid beta (Aβ), may create an optimal environment for the pathogenesis of AD (Fig. 1E)^[^71^]^. Primary injuries are unavoidable mechanical traumas, whereas secondary injuries evolve due to neuroinflammatory cascades, providing a therapeutic window. Understanding microglial involvement is crucial for developing targeted interventions. This review highlights the role of microglia in secondary TBI pathophysiology and explores therapeutic strategies to modulate neuroinflammatory responses, aiming to improve patient outcomes.

## The critical roles of microglia in TBI

Immediately after TBI, an immune response is triggered, and inflammation can fulfill both protective roles in the healing process and pathological roles in sustaining the injury^[^72^]^. When immune-regulated recovery and compensatory mechanisms proceed without disruption, there is a good chance of functional improvement following a mild, isolated CNS injury. Nonetheless, CNS injuries do not always resolve quickly; multiple factors, including age, genetic predisposition, previous injuries, and infections, may hinder this process^[^73^]^. Owing to the constant disruption of CNS barriers, the brain parenchyma exists in a chronic state of immune activation and imbalance. This delay in immune restoration fosters the neurodegenerative processes. After an initial injury, neuroinflammation is perceived as vital, potentially worsening the damage itself or facilitating the growth and development of neural stem cells (NSCs)^[^67,74^]^. Establishing the specific role of inflammation in neurogenesis and neural regeneration presents a significant challenge for researchers. Although inflammation can be advantageous during the initial phases, it has the potential to cause harm and contribute to neurodegeneration in the long term. Gaining insight into the underlying mechanisms of neuroinflammation, considered to perform a dual role, and leveraging its ability to promote neurogenesis offers a promising strategy for tackling this clinical challenge^[^75^]^.

Microglia comprise approximately 5-15% of the total brain cell population^[^76^]^. Microglia are resident immune cells that act as sentinels in the CNS. Within the CNS, microglia are vital for brain development, synaptic plasticity that is dependent on activity, and adult learning. They regulate key functions, such as neuronal surveillance, neurogenesis, cell death, and synapse elimination^[^77-80^]^. Microglia react to TBI within minutes of injury. This is evidenced by the projection of processes toward the injury site, closely followed by morphological changes and proliferation^[^81,82^]^. In response to tissue injury, these cells activate a two-fold approach that includes amplified inflammatory responses to dispose of damaged cells, engage in phagocytosis, and secrete neurotrophic factors to mend and rejuvenate compromised structures (Fig. 1A) ^[^83^]^. In the resting state, microglia show a ramified morphology featuring small cell bodies and relatively long cytoplasmic projections that extend into the environment, continuously assessing neuronal activity and the surrounding brain microenvironment^[^84,85^]^. Upon exposure to stimuli such as injury, illness, or pathogens, microglia adopt a polarized activation state. Upon activation, these cells become motile, abandon their branched structure, and adopt an amoeboid shape with a larger cell body and no retracted projections, resembling peripheral macrophages^[^83,86^]^. As expected, microglia demonstrate a swift and vigorous response to injury, as indicated by imaging studies conducted within 24 h^[^87^]^. Homeostatic genes such as *P2ry12, Tmem119*, and *Tgfbr1* show reduced expression, while genes involved in RNA and protein synthesis, inflammatory responses, phagocytosis, lipid metabolism, and cytokine synthesis are upregulated^[^88-90^]^. Evidence indicates that the microglial response occurring within minutes to hours after an injury is beneficial and contributes to the containment of CNS lesions^[^91-93^]^. In the immediate aftermath of an injury, microglia play a crucial role in restoring the structural integrity of the compromised BBB by extending their processes and surrounding blood vessels^[^94^]^. Subsequently, microglia are recruited to clear necrotic debris and engage in phagocytosis via purinergic signaling (Fig. 1B) ^[^81,95,96^]^. Microglia actively contribute to the clearance of dead cells from the parenchyma and are crucial for preserving the integrity of CNS barrier structures, including glial limitans and blood vessels^[^81,97^]^. In a reactive state, microglia are incapable of fulfilling the barrier closure function^[^87^]^. It is noteworthy that the microglial response was inadequate when mTBI occurred again within 24 h of the initial injury, leading to heightened leakage at the superficial glial cell boundary and increased neuronal death in the cerebral cortex^[^72^]^. The inability of reactive microglia to serve as a barrier seal following injury increases the susceptibility of the CNS to secondary damage^[^87^]^. Apart from their function as barriers, microglia are also involved in synthesizing neuroprotective factors, clearing away cellular debris, and facilitating nerve structural restoration processes, which are essential for neurological recovery after TBI^[^77,98,99^]^. After injury, the microglial population displays a balanced distribution between pro-inflammatory and anti-inflammatory states during the first week. This process facilitates neurogenesis and augments the immune response to injury (Fig. 1C)^[^100-102^]^. Alongside the primary focal response, activated microglia have been found in remote areas, such as the thalamus and hippocampus, present on both the same and opposite sides of the injury, observable as early as 7 days following TBI in mouse models^[^103^]^. Brain imaging studies and postmortem tissue examinations have shown that microglial cells exhibit increased levels of activation markers, such as CR3/43 (major histocompatibility complex class II (MHCII)), CR3, CD68, and PK11195, approximately 17 to 18 years after an injury, particularly in cases of isolated blunt head trauma and repeated impacts. This phenomenon has been documented in those who have sustained various high-impact events during their sporting careers, like retired professional football players^[^66,104,105^]^. Further characterization of CD68^+^/MHCII ^+^ microglia in animal studies has indicated that they display a primed state. In this primed state, microglia exhibit a more intense inflammatory profile and are more prepared to face subsequent challenges^[^106^]^. This early response of microglia restricts lesion expansion. However, this response also results in the production of inflammatory cytokines and reactive oxygen species (ROS), which are associated with negative effects on recovery, and may result in secondary injury if proper resolution does not occur^[^107^]^. The dual role of microglia in promoting neuroprotection and inducing inflammation highlights the complexity of their functional dynamics after injury. As time elapses after injury, the neuroprotective functions of microglia diminish, and they become the primary regulators of neuroinflammatory gene expression in the post-TBI environment^[^108^]^. Chronic activation of microglia can become dysregulated, leading to an increased production of pro-inflammatory and cytotoxic mediators that impair CNS tissue structural restoration, ultimately causing neuronal damage and cell death^[^77^]^. Extended dysregulation of microglia and neuroinflammation have been shown to be harmful to the CNS^[^109^]^ (Fig. 1D).

### Polarization of the microglia after TBI

The standard classification of activated microglia identifies various phenotypic subtypes based on their morphological features, surface marker expression, and physiological characteristics^[^110^]^. It has been suggested that the M1-M2 classification can be used to identify microglial activation in experimental systems, differentiating between the conventional M1 and alternative M2 phenotypes^[^111^]^. In recent years, the advent of neuroinflammatory research has led to a more comprehensive study of microglia. Consequently, there is a growing consensus advocating the abandonment of the M1/M2 typing system in favor of a more dynamic approach for studying microglial function. This approach involves combining technical knowledge from fields, such as transcriptomics, proteomics, and single-cell sequencing, to observe and describe changes in microglia throughout the inflammatory response. This shift emphasizes the necessity for a more detailed understanding of microglial behavior, its implications for neuroinflammatory conditions, and potential therapeutic interventions^[^112^]^. However, given the extensive body of literature examining the M1 and M2 phenotypes, we have undertaken a concise categorization and synthesis of the current understanding of these classifications.

Previous investigations have illustrated the significant role of the microenvironment surrounding the injury site in determining how microglia and macrophages polarize in response to CNS damage. Extracellular signals present in this biochemical environment initiate phenotypic changes and influence cellular responses^[^113^]^. The induction of the M1-like phenotype is associated with several essential mechanisms, including phagocytosis, pathogen eradication through iron limitation, acidification of phagosomes, and ROS generation^[^113-115^]^. In numerous situations, the M1-like response proves advantageous and is usually downregulated following the elimination of harmful agents or pathogens; on the other hand, uncontrolled or excessive activation of this response within the brain might induce the secretion of pro-inflammatory compounds and neurotoxic mediators, leading to cycles of neurodegeneration induced by microglia^[^116,117^]^. LPS, interferon gamma (IFN-γ), and GM-CSF are the primary mediators of M1 polarization^[^118-120^]^. Nonetheless, various experimental and clinical studies have indicated that a single moderate TBI or several mild TBIs can lead to an enduring M1-like phenotype that lasts months to years. This phenotype correlates with impaired structural restoration of neural tissue, particularly after moderate-to-severe TBI^[^96,98,121-125^]^. Various soluble factors found in the microenvironment of lesions, including tumor necrosis factor-alpha (TNF-α), IFN-γ, and lipocalin-2, have been recognized as significant drivers of the transition from M2 to M1 macrophages^[^126,127^]^. This response is essential for the host defense. Certain markers, including CD16, CD32, CD86, MHCII, and iNOS, define the M2-like phenotype. In response to pro-inflammatory signals, including IFN-γ, both macrophages and microglia evolve into a “classical” M1-like phenotype, distinguished by enhanced production of pro-inflammatory cytokines [interleukin-6 (IL-6), IL-1β, IL-12, TNF-α], chemokines (CCL2, CXCL9, CXCL10), and ROS. This mechanism is crucial for host defenses^[^113,115,128^]^. Understanding the complexities of microglial polarization in the CNS is essential for the development of targeted therapies aimed at modulating inflammatory responses after injury. M2 cells were localized in the perilesional area, whereas M1 cells were predominantly located within the lesion itself. This research reinforces the notion that the M1 response is linked to the removal of necrotic tissue, whereas the M2 response preserves salvable tissue in the perilesional area. These transitional phenotypes have been demonstrated to exert either beneficial or detrimental effects, contingent upon the stimuli, their duration, and the environment in which they operate^[^101,129,130^]^. The M2-like reaction is temporary, with a clear shift towards an M1-like response detectable within a week after trauma. Nevertheless, the M2-like reaction lasts for only a short duration, as a shift towards a predominant M1-like response can be seen within a week after the injury occurs^[^131,132^]^.

For tissues to heal successfully following TBI, the contributions of both M1-like and M2-like responses are likely essential, as they must undergo synchronized transitions to effectively govern the series of inflammatory, proliferative, and remodeling processes vital for healing^[^133^]^. Alternatively, adjusting the balance between various phenotypes may be more effective in fostering optimal remodeling of the CNS. This idea is reinforced by recent experimental studies that highlight the benefits of modulating M1/M2-like polarization to prevent neurodegeneration following trauma and promote CNS remodeling^[^113^]^. Therefore, while the M1-like response is crucial for early recovery, a premature transition to the M2-like state may have detrimental effects. Thus, therapeutic strategies must be precisely timed to prevent chronic immunosuppression and maladaptive processes (Fig. 1).

### Role of microglia in the neuroinflammation of TBI

Neuroinflammation is a hallmark of TBI, with microglia serving as central regulators of this process. Their activation is governed by a complex interplay of intrinsic and extrinsic signals, which collectively shape the inflammatory response and influence injury outcomes. This section explores the key regulatory mechanisms underlying microglia-mediated neuroinflammation. We first discuss extrinsic signals, particularly damage-associated molecular patterns (DAMPs), which modulate microglial activity in response to injury. We then examine intrinsic factors, including cytokine-related pathways and membranous receptor-mediated signaling. Finally, we highlight the intricate crosstalk between intrinsic and extrinsic pathways, focusing on key signaling molecules and inflammasomes that integrate these regulatory mechanisms. Understanding these interactions provides critical insights into the microglial response in TBI and informs potential therapeutic strategies.

### Extrinsic signals in regulating inflammation

After cell death occurs, signals referred to as alarmins and DAMPs are emitted into the extracellular space, notifying innate immune cells, such as microglia^[^134,135^]^.

### DAMPs

Following TBI, DAMPs are released by injured neurons, glial cells that gather and process various signals within the brain, and immune cells that infiltrate the injury site^[^136^]^. These DAMPs include ATP, high-mobility group box 1 (HMGB1), and other molecules^[^81,137,138^]^. DAMPs modulate the activation and function of microglia through several mechanisms, consequently promoting inflammatory responses. These signals not only intensify localized inflammation but may also impact neuronal recovery by regulating processes associated with cell survival and recovery. A thorough understanding of the roles and functions of these DAMPs could offer significant insights into the therapeutic strategies designed to improve recovery after TBI.

**HMGB1:** As an important cytokine in the inflammatory process, HMGB1 may represent a potential therapeutic target in TBI. It enhances the release of additional cytokines, engages microglia, and aggravates neuronal injury^[^139,140^]^. HMGB1 is secreted by monocytes and macrophages and expressed in the cytoplasm of microglial cells^[^141^]^. It has also been proposed that cellular injury or death during TBI may cause HMGB1 to relocate from the nucleus to the extracellular space. This translocation may result in microglial activation and subsequent release of HMGB1^[^142^]^. It has been hypothesized that glycyrrhizin can alleviate the severity of TBI by inhibiting M1 phenotype activation and facilitating M2 phenotype activation in microglia, at least partly through HMGB1 inhibition^[^143^]^. The mechanism by which HMGB1 and GL inhibit macrophage and microglial polarization may involve HMGB1 receptors, including the receptor for advanced glycation end products, Toll-like receptors (TLRs), including TLR2 and TLR4, and the downstream nuclear factor kappa B (NF-κB) pathway^[^144^]^.

**Figure 3.**
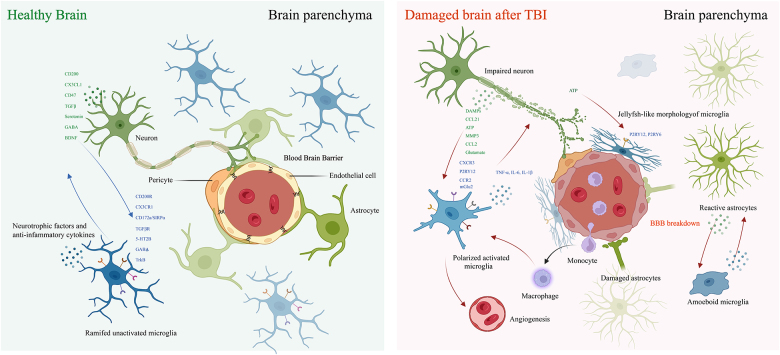
The crosstalk between microglia with other cell types in TBI. The BBB comprises various cell types, including endothelial cells, astrocytes, and pericytes. In a healthy brain, resting microglia continuously monitor the brain’s microenvironment and interact with diverse brain cells, thereby maintaining homeostasis under physiological conditions. For example, neurons release inhibitory signals targeting microglia, which helps reduce excessive activation and phagocytic activity of these cells. Following TBI, (1) Crosstalk Between Microglia and Neurons: At the injury site, compromised neurons release substantial amounts of ATP, cellular debris, EVs, and various mediators that collectively activate microglia, including the initiation of microglial activation, phagocytic activity, and recruitment. Microglia secrete early pro-inflammatory factors such as TNF-α, IL-6, and IL-1β. These inflammatory mediators stimulate neurons to release ROS, ultimately resulting in axonal damage. (2) Crosstalk Between Microglia and Astrocytes: Peri-lesional astrocytes exhibit reactivity by increasing GFAP and producing cytokines to recruit microglia and peripheral immune cells. When microglia detect ATP via the P2RY12 receptor, they swiftly extend their processes to close the gaps between astrocytes. However, when the expression of P2RY12 on microglia decreases, their sealing capacity is diminished, potentially driving astrocytic toxicity during inflammation. (3) Crosstalk Between Microglia and Macrophages: Early monocytes play a vital role in angiogenesis by bestowing pro-angiogenic properties on microglia, such as improving their ability to produce VEGF. Subsequently, infiltrating macrophages inhibit microglial activation by decreasing the expression of inflammatory molecules and their phagocytic capacity, thereby preventing chronic microglia-mediated inflammation in the CNS. Depending on the extent and severity of the injury, the intricate interactions among various cell types may be detrimental, resulting in excitotoxicity, neuroinflammation, demyelination, apoptosis, and other complications; alternatively, they may be neuroprotective, facilitating repair and survival. (Image created with BioRender.Com).

**ATP:** Purinergic receptors represent an evolutionarily ancient family of transmembrane molecules that can detect ATP, ADP, and adenosine^[^145,146^]^. These receptors are categorized into two primary classes: P1 receptors, which react to adenosine, and P2 receptors, which react to ATP and ADP. ATP functions as a cellular energy source and is maintained at elevated intracellular concentrations under steady state conditions. Following tissue injury, ATP is released from injured cells, subsequently initiating an immune response through purinergic receptor signaling^[^97^]^. Following brain injury, ATP release initiates a microglial response via purinergic signaling. This purinergic signaling system is crucial for regulating neuroinflammation and can notably alter the overall response of the CNS to injury. Microglia are activated by fluctuations in ATP levels outside of the cell via multiple purinergic receptors. These cells contain P2Y receptors that couple with G-proteins and P2X receptors that interact with ligand-gated cation channels^[^147^]^. The presence of purinergic receptors in microglia is marked, and they are vital for several key functions including cytokine production (through P2X receptors), cell movement (mediated by P2Y12 receptors), and phagocytosis (via P2Y6 receptors)^[^148,149^]^. Purinergic receptor signaling leads to responses that vary and depend on factors such as the dynamics of receptor expression, the involved immune cell types, the specifics of the injury, and the duration since the injury occurred. For instance, purinergic receptor signaling has a significant impact on primary activation and shape changes of microglia following laser-induced mTBI^[^81,95,150^]^. Blocking purinergic receptors to antagonize early microglial responses has been associated with a higher incidence of damage in models of mTBI^[^81,97^]^. A previous study demonstrated that the release and detection of ATP through purinergic receptors elicits an acute neuroprotective inflammatory response following mild cortical injury^[^81^]^. Several recent studies have reported an increase in inflammasome activity following TBI, primarily in activated microglia. The multifaceted roles of purinergic signaling in modulating microglial function reveal promising routes for the treatment of neuroinflammation and recovery from brain injury. In response to brain injury, microglia extend their processes toward glial limitans and encircle individual astrocytes, forming a hexagonal honeycomb structure. This response is mediated by purinergic receptor signaling through P2X4 and P2Y12, as well as ATP-dependent release from astrocytes via connexin hemichannels^[^134^]^. Research indicates that Microglia express P2RY6, a UDP receptor critical for phagocytosis^[^148^]^. Following mTBI in mice, the death of glial limitans astrocytes leads to increased activation of microglia in response to P2RY6 signaling. This transformation allows microglia to adopt a jellyfish-like shape, enabling them to occupy the spaces created by dying astrocytes, leading to the uptake of debris^[^81^]^. Using a focal cortical contusion model, researchers found that inhibition of this receptor hampers the ability of microglia to become phagocytes, resulting in a higher number of necrotic cells present within the neocortex^[^81^]^. P2X7 receptor (P2X7R) is mainly localized in microglial cells of the cerebral cortex and shows heightened expression levels after an episode of TBI. Following injury, microglia evolve into amoeboid-like cells, emitting many microvesicle (MV)-like particles throughout damaged and adjacent tissues. The combination of a P2X7R blocker (A804598) and immunosuppressant (FTY720) produced a significant decrease in MV-like particles in the affected and adjacent areas, including the CSF. Moreover, the frequency of apoptotic neuronal death was reduced, while the neuronal survival rate in the injured and nearby cerebral cortex regions was increased^[^81^]^. It is imperative to investigate the distinct roles of various purinergic receptor types at different stages of TBI (Fig. 3).

**S100B:** S100B is a member of the S100 family, characterized by its small cytoplasmic proteins that bind calcium, and was first characterized by Moore in 1965^[^151^]^. Treatment with neutralizing antibodies against S100B in mice can reduce lesion volume caused by TBI, attenuate microglial activation, improve neuronal survival, enhance memory function, and diminish sensorimotor deficits^[^152^]^. At low concentrations, S100B functions as a neurotrophic factor; however, excessive production by activated glial cells can exacerbate neuroinflammation and neuronal dysfunction^[^153^]^.

### Intrinsic signals in regulating neuroinflammation

The intrinsic characteristics of microglia, such as their specific gene expression profiles, altered metabolic states, and enhanced receptor sensitivity, significantly influence their functional outcomes during the inflammatory process. Activated microglia can secrete pro-inflammatory cytokines such as TNF-α, IL-1β, and IL-6, which may contribute to an excessive inflammatory response, potentially leading to neuronal and glial cell damage. The shift towards a reparative state is influenced by various intrinsic factors, including gene regulation and the activation of specific intracellular signaling pathways. These factors enable microglia to adapt their functional characteristics in response to environmental signals and molecular cues. The dynamic interplay between pro-inflammatory and anti-inflammatory signals underscores the importance of feedback mechanisms in modulating microglial responses and shaping outcomes following TBI.

### Cytokines related pathways

The expression of inflammatory factors occurs quickly after TBI, facilitating the coordination of both local and peripheral immune cell activities. These mediators are pivotal in orchestrating the inflammatory response and ultimately shaping the results of injury and recovery. The following section summarizes the main mediators involved in this process.

**IL-1β:** The damage to cellular membranes caused by primary mechanical injury or secondary trauma results in the release of DAMPs^[^134,154,155^]^. Local glial cells and infiltrating immune cells significantly elevate the levels of TNF, IL-6, and IL-1β, acting as initial effectors that play a crucial role in post-traumatic neuroinflammation^[^156^]^. The IL-1 family of cytokines is crucial in influencing the generation of other cytokines, aiding phagocytosis, and inducing cell death through programmed mechanisms^[^157^]^. IL-1 has been recognized for its neuroprotective abilities; however, specific isoforms, such as IL-1β, or sustained high levels of IL-1 have been linked to acute neurodegenerative processes, reduction in neurogenesis, and induction of cell death^[^158^]^. As discussed earlier, IL-1β and IL-18 arise from inflammasomes and are often detected during sterile injury responses. For example, IL-1β levels in the cerebrospinal fluid (CSF) of patients suffering from severe TBI are elevated for a minimum of 24 h before they begin to drop, and this is linked to increased intracranial pressure and poorer clinical outcomes^[^159,160^]^. It is generally accepted that IL-1β stimulates the growth of macrophages and neuroinflammatory cells, including microglia and astrocytes^[^161^]^. In cases of mTBI, neuroinflammation may self-resolve depending on the severity of the injury. However, in more severe TBI scenarios, persistent or excessive neuroinflammation is marked by the production of cytokines such as IL-1β, which can lead to the proliferation and accumulation of immune cells at the injury site, including microglia, macrophages, and astrocytes^[^162^]^. A further clinical study demonstrated a robust link between IL-1β concentrations and sustained neuronal injury at the six-month mark following TBI^[^163^]^. Animal experiments have revealed a detrimental role for IL-1β, indicating that treatment with an IL-1 receptor antagonist reduces lesion volume in TBI and improves neurological performance^[^164,165^]^. Research utilizing a mouse TBI model indicated that blocking elevated IL-1β levels in the globus pallidus results in diminished activation of microglia, thereby alleviating the extent of damage incurred from FPI^[^166^]^. These findings underscore the importance of targeting IL-1β for therapeutic strategies aimed at mitigating the long-term effects of TBI.

**IL-18:** The engagement of both IL-1β and IL-18 by a protein complex that forms inflammasomes may become excessive, resulting in increased production of these cytokines and possibly influencing the pathology of disorders, such as AD^[^167^]^, multiple sclerosis (MS), and TBI^[^162^]^. Treating mice with an IL-18 antagonist 1 h after injury enhanced their neurological recovery when administered as a therapy^[^168^]^. These data indicate that both IL-1β and IL-18 contribute to the exacerbation of TBI-related mechanisms in models of cerebral bruising^[^162^]^.

**IL-6:** IL-6 was increased after TBI. Nevertheless, this cytokine can have both beneficial and detrimental effects^[^169^]^. The level of IL-6 found in the CSF correlates with positive outcomes in children with severe TBI^[^169^]^. As a significant mediator in the inflammatory process, IL-6 has been acknowledged to assist in the rapid defense of tissues from damage through immune system activation^[^170^]^. IL-6 plays two distinct roles in the post-injury inflammatory response and has been established as an early indicator of the severity of TBI^[^171^]^. The concentration of IL-6 was found to increase significantly within 6 h and remained elevated for up to 6 months following mTBI^[^172,173^]^. Findings from mouse studies reinforce this observation, as they reveal that the absence of IL-6 hampers recovery from TBI, whereas introducing IL-6 transgenically in astrocytes promotes the healing process through a mechanism believed to assist revascularization at the injury site^[^174^]^. IL-6, recognized as a neuroprotective cytokine, has been demonstrated to shield neurons from damage after injury by promoting the proliferation of microglia at the site of injury^[^175^]^. IL-6 has occasionally been identified as a neurotoxic cytokine. Elevated plasma levels of IL-6 following severe TBI are associated with negative long-term clinical outcomes, particularly cognitive dysfunction^[^176^]^. Serum levels of IL-6 have been linked to the onset of multiorgan failure, sepsis, and adverse neurological outcomes in patients with severe TBI^[^177^]^. Extracellular IL-6 can induce brain edema, disrupt the integrity of the BBB, and cause neuronal damage, contributing to pro-inflammatory effects. However, IL-6 may also have an anti-inflammatory effect at the site of injury. The effects of IL-6 on microglial activation and its influence on neuronal recovery or degeneration following TBI are dependent on the acute or chronic phase of injury^[^175^]^. This suggests that therapeutic strategies targeting IL-6 should consider the time elapsed since the injury.

**TNF-α:** As a transmembrane protein, TNF-α is created and can be cleaved from the membrane by the TNF-α converting enzyme (also known as ADAM17), resulting in a soluble cytokine. It can then be released by microglia, astrocytes, and neurons^[^178^]^. The neurogenic effects of both pro- and anti-TNF-α have been observed in various models, methodologies, and cell types^[^178^]^. TNF-α concentrations were found to be higher in individuals diagnosed with TBI^[^179-181^]^. Within 4 h of experiencing diffuse brain injury, mice exhibited a short-lived elevation in the expression of IL-1β, TNF-α, and CD14 in the cortex and hippocampus 72 h after the injury, and these molecule levels returned to baseline^[^182^]^. Notably, TNF-α-knockout mice experienced significantly less neurological impairment 7 days after TBI. However, at the 2- to 4-week mark, a reverse trend emerged, with TNF-α-deficient mice displaying more extensive lesion formation and increased motor dysfunction compared to wild-type (WT) controls^[^183^]^. These findings emphasize the multifaceted role of TNF-α in post-injury recovery, suggesting that, while it has neuroprotective effects in the short term, its prolonged absence may lead to adverse outcomes. Notably, the combined genetic inhibition of TNF-α together with the Fas receptor led to a reduction in tissue damage and superior functional outcomes following CCI. Nevertheless, utilizing a mouse model of concussion marked by impact and head acceleration without any structural brain injury results in worse functional outcomes with this strategy^[^184,185^]^. These results highlight an important principle that various molecular mechanisms can execute distinct functions depending on the pathoanatomical context of TBI. Moreover, these results indicate that targeting TNF-α/Fas may offer benefits to those with cerebral contusions, while it might pose risks to individuals with concussions^[^82^]^. These findings reveal that TNF-α serves a dual purpose in the aftermath of cerebral contusion, contributing to acute pathogenesis as well as enhancing tissue remodeling and neurological improvement. These results emphasize the intricate nature of targeting inflammatory pathways, as the therapeutic outcomes of TNF-α modulation can vary significantly based on the specific type of brain injury and timing of the treatment intervention. Following diffuse TBI, microglia were found to be the key source of TNF-α^[^186^]^.

**IL-10:** IL-10 is a strong anti-inflammatory agent that works through the STAT3 pathway^[^160,187,188^]^. Clinical research has indicated that IL-10 levels increase during the first 3 h after TBI, with the possibility of this elevation lasting for up to 6 months. This statement suggests that IL-10 may be essential in mitigating an increased pro-inflammatory burden, as reflected by the higher concentrations of TNF-α and estradiol detected in the serum of patients following severe TBI^[^189-191^]^. In patients with TBI, it is detected at higher concentrations in the CSF and is associated with circulating monocytes^[^160,187,188^]^. IL-10 plays a significant role in mediating interactions between microglia, astrocytes, and neurons^[^192^]^. In vitro research has demonstrated that TLR activation in microglial cells leads to the production of IL-10. This response has been detected in microglial cells activated by TLR2, TLR3, TLR4, and TLR9^[^193-197^]^. IL-10 suppresses the generation of pro-inflammatory cytokines by microglia, thereby shielding astrocytes from the harmful consequences of excessive inflammation^[^194^]^. Timely administration of IL-10 after injury provides neuroprotective benefits. It has been postulated that IL-10 might influence the immune response located in the periphery to exert its effects^[^82^]^. These insights into the impact of IL-10 underscore its crucial role in neuroprotection and inflammation resolution after TBI, suggesting that strategies to enhance IL-10 signaling could have therapeutic potential. The maintenance of both the brain and peripheral tissues after TBI relies on anti-inflammatory factors, such as IL-10. In normal tissue settings, this cytokine is essential for preserving vascular stability by modulating oxidative stress and inflammation. This ultimately protects against potentially damaging effects that could otherwise occur in the vascular and endothelial systems^[^198^]^. In TBI experimental setups, the application of IL-10 has been shown to decrease the size of brain lesions, fluid accumulation, and injury-related cognitive issues. These favorable outcomes involved an enhancement in cell viability and preservation of BBB integrity, especially when utilized alongside hyperbaric oxygen treatment. Treatment with IL-10 resulted in less damage, whereas the naturally raised IL-10 levels noted in TBI patients were associated with increased mortality rates^[^199^]^. These findings underscore the dual role of IL-10 in TBI, highlighting its promise as a treatment modality for mitigating damage and as a marker of inflammatory response severity in patients.

**Transforming growth factor-β (TGF-β)**: TGF-β is a significant cytokine that possesses various immune regulatory roles and has been recorded in the CSF of patients diagnosed with TBI^[^187,200^]^. In the CNS, different isoforms of TGF-β (TGF-β1, TGF-β2, TGF-β3) and their related receptors are found in neurons, astrocytes, and microglial cells during instances of brain injury and neurodegenerative diseases^[^201,202^]^. Utilizing a small hairpin RNA technique to reduce TGF-β1 levels in the contused region resulted in a dramatic increase in neuronal cell death, substantial reduction in astrogliosis, and worsening of neurological deficits. These findings indicate that TGF-β1 provides neuroprotection and enhances astrogliosis in a rat TBI model. An investigation into macrophage involvement in TBI-related tissue restoration found that administration of TGF-β increased the emergence of a particular subtype of macrophages associated with tissue remodeling. The macrophages demonstrated a gene expression profile akin to that of microglia, cells located in the brain which have functions in neuroprotection, angiogenesis, and cellular migration. These observations indicate that insufficient TGF-β may be a factor in unfavorable neurological prognoses^[^203^]^. In a different study that examined the functions of bone morphogenetic protein (BMP) and TGF-β in TBI, the observed microglial response was linked to TGF-β. Conversely, the combination of TGF-β and BMP facilitates the regeneration of the BBB and reactivation of astrocytes^[^204^]^. TGF-β exerts beneficial effects after TBI. Inhibition of ADAM17 results in elevated levels of TGF-β receptors expressed on the membrane of microglia and enhances the formation of TGF-β1/TGF-βR complexes. This is accompanied by the nuclear translocation of Smads, activation of the TGF-β1/Smad pathway, and promotion of the M2 phenotype in microglia, ultimately exerting neuroprotective effects^[^205^]^. These insights into the role of M2-like microglia and TGF-β highlight their potential as therapeutic targets for optimizing the recovery processes following TBI.

**Interferon (IFN):** Single-cell RNA sequencing performed on the cortex 7 days post diffuse TBI revealed distinct microglial clusters characterized by type I IFN gene expression. The expression levels of genes related to IFN signaling pathways increased 7 days post-injury (dpi), coinciding with an increase in microglia exhibiting primed profiles^[^108^]^. In addition, a number of investigations have revealed that type I IFN signaling is crucial for the change from acute to chronic phases in adult mice after both diffuse and focal TBI^[^206,207^]^. In fact, microglial *IFNAR1* mRNA expression is higher than that of other cell types in the CNS^[^208^]^. For instance, *IFNβ* mRNA levels are markedly elevated in individuals who succumb to their injuries shortly after TBI^[^209^]^. The antiviral effects triggered by IFNs show significant correlations with a variety of genes that are stably expressed in microglial cells following TBI, including those related to *MHCII, CD11c* and *CD68*^[^210^]^. A study utilizing CCI demonstrated that the transcriptomes of both microglia and astrocytes exhibited enrichment of type I IFN responses at 7 dpi^[^211^]^. The function of IFN in secondary injury after TBI was examined using a regulated cortical impact model in adult male mice lacking *IFN*-(*IFN^-/-^*). Following TBI, an increase in pro-inflammatory mediator expression was observed in the cortex and hippocampus of WT mice, while this elevation was reduced in *IFN^-/-^* mice. Furthermore, sustained activation of microglia and deficits observed in motor and cognitive functions were diminished in *IFN^-/-^* mice with TBI as opposed to their injured WT counterparts. The improved neurological recovery observed in *IFN^-/-^* mice is linked to smaller lesion volumes and decreased neurodegeneration in the hippocampus. Microglia are essential to produce and response to IFNs in the context of TBI. IBA-1^+^ meninges macrophages may also serve as an additional source of IFN and have been noted to show increased type I IFN gene expression after mTBI^[^212^]^. Collectively, these findings underscore the critical role of IFN signaling pathways contributing to the inflammatory response and enhancing recovery in the context of TBI. Elevated IFN signaling has been linked to the harmful neuroinflammation observed in both adult and aged cohorts after TBI. Furthermore, this process may influence long-lasting priming of microglia. Upon triggering by DAMPs, free radicals, and pro-inflammatory cytokines such as IFN-γ, microglia adopt a phenotype similar to that of M1 microglia^[^113^]^. When stimulated by IL-4 or low doses of IFN-γ, microglia that possess M2-like traits release neurotrophic factors, including insulin-like growth factor-1 (IGF-1), consequently aiding neurogenesis^[^213^]^. Administration of PLX5622 prior to the experimental injury prevented the regulation of inflammatory genes in response to various stimuli, IFNs, and neuronal synaptic damage. This was accompanied by a significant reduction in cognitive impairment at 7 and 30 dpi^[^108^]^. Additionally, IFNβ signaling observed within the first 24 h after CCI is significant in instigating downstream inflammation, tissue injury, and deficits in function^[^207,214^]^. Another study found that type I IFN mediators, including pSTAT1, Irf7, and cyclic GMP-AMP synthase (cGAS), were elevated 24 h post-CCI in elderly mice^[^215^]^. Collectively, these results emphasize the multifaceted function of IFN signaling in influencing inflammatory responses and neurodegeneration following TBI. The signaling pathways of type I and II IFN were markedly increased in older mice after TBI compared to those in adult mice. A wide range of central mediators in IFN signaling, such as IFNAR1, IFN-α/β/γ, STING1, IRFs 1/3/7, and Stat1, showed increased expression in aged mice with TBI compared to their adult counterparts^[^216^]^. Research indicates a connection between IFN signaling and poor prognosis following diffuse TBI in aged mice^[^216^]^. IFN signaling following injury may present a promising avenue for therapeutic intervention aimed at mitigating persistent neuroinflammation, particularly in elderly individuals^[^216^]^. STING, known as the stimulator of IFN genes, plays an essential role in mediating IFN responses following TBI by functioning as a protein that responds to stress within the endoplasmic reticulum. It is associated with cyclic cGAS functions to detect tissue damage, extracellular DNA, and mitochondrial irregularities, which promote the production of type I IFN genes^[^217^]^. Further research has demonstrated that STING and cGAS are expressed in microglia within the brain^[^206,214^]^. One study demonstrated a reduction in IFN-I signaling following TBI using STING-deficient mice and STING antagonists, particularly chloroquine. After TBI, STING levels were greatly decreased in STING KO mice, along with changes in the morphology of microglia and a reduction in the expression of inflammation-associated genes (*Tnf, Cd68, Ccl2*) and IFN genes (*Irf3, Irf7, Ifi27*) within the cortex. In addition, CQ has been demonstrated to counteract cognitive impairments following TBI and significantly reduce the expression of inflammation-related genes (*Cd68, Tnf, Ccl2*) along with IFN genes (*Sting* and *Irf7*) within the cortex^[^218^]^. Repeated intraperitoneal doses of the STING agonist DMXAA significantly amplified type I IFN signaling in the brain, which led to increased microglial restructuring in the cortex and hippocampus 7 days post-treatment. DMXAA enhanced the levels of various inflammatory genes in the cortex of adult mice after TBI. Consequently, enhanced type I IFN signaling post-TBI leads to increased microglial activation in the cortex at 7 dpi^[^216^]^. The augmentation of IFN signaling through the application of the STING agonist DMXAA in conjunction with TBI resulted in exacerbated cortical inflammation and microgliosis in adult mice. Similarly, following diffuse TBI, the boost in IFN signaling observed in older brains is related to persistent cortical inflammation and gliosis^[^216^]^. A reduction in IFN-I signaling following TBI through STING-dependent interventions has been observed to attenuate prolonged microglial activation and cognitive impairment^[^218^]^. These findings suggest that an enhanced type I IFN signaling pathway is associated with detrimental neuroinflammatory responses in both adults and older adults following TBI. This might have contributed to the prolonged microglial activation observed in these individuals. Therefore, targeting the IFN pathway may be a promising therapeutic approach for TBI.

### Membranous receptor mediated pathway

**TLR:** TLRs are located at the leading-edge constitutive elements of the innate immune system and are essential for the activation of immune responses. TLRs are responsible for identifying pathogens and DAMPs and serve as key receptors for microglia in the detection of pathological states. Microglia are unique among innate immune cells within the CNS because of their high expression of TLRs, particularly TLR4^[^219^]^. TLR4 signaling plays a harmful role in the pathology of TBI^[^220,221^]^. MicroRNA-124 (miR-124) is a miRNA that is specifically found in the brain and is highly abundant in microglial cells. Exposure to miR-124-enriched exosomes (Exo-miR-124) promotes microglial M2 polarization, enhances hippocampal neurogenesis, and increases functional recovery after TBI. The M2 polarization action of Exo-miR-124 is mediated by the inhibition of the TLR4 signaling pathway^[^222^]^.

**Chemokine receptor:** CX3CL1, also known as fractalkine, possesses the characteristics of both chemoattractants and adhesion molecules, making it the sole member of the CX3C (delta) subfamily of chemokines. CX3CL1 interacts specifically with its receptor, CX3CR1, which is expressed in microglia. Under physiological conditions, microglia utilize tonic signaling via the CX3CL1/CX3CR1 axis, aiding the maintenance of a quiescent state and supporting homeostasis within the neuronal network^[^223^]^. Concerning neurological outcomes, it is noteworthy that *CX3CR1^−/−^* mice exhibited superior performance compared with WT mice in the neuroscore test conducted on day 4 post-TBI. Nevertheless, it is crucial to recognize that their performance declined during Week 5. Inhibition of the CX3CL1/CX3CR1 axis has been shown to provide beneficial effects shortly after TBI. Nevertheless, the long-term outcomes are less favorable^[^224^]^. An additional study indicated that CX3CR1 knockout exacerbated WM damage three days post-TBI, with this damage persisting for at least 28 days. Three days post-injury, the absence of CX3CR1 did not affect lesion volume or brain water content. Nevertheless, it significantly diminished cell survival and increased cell death near the lesion. CX3CR1 knockout mice demonstrated increased cell death around the lesion, likely due to the deficiency, CX3CR1 impacting microglial migration to the lesion site and its anti-inflammatory effects^[^225^]^. Research indicates that although *CX3CR1^−/−^* mice demonstrate improved short-term neuroscore performance following TBI, long-term outcomes are detrimental, underscoring the complex role of the CX3CL1/CX3CR1 signaling pathway in TBI recovery^[^225^]^. In conclusion, it can be stated that *CX3CR1^−/−^* mice exhibited enhanced short-term neurological outcomes; nevertheless, they experienced exacerbated long-term brain damage, which may be attributed to increased cell death and impaired microglial migration.

**P2Y12R:** P2Y12R is a microglia-specific receptor This receptor mediates microglial chemotaxis toward the injury site^[^226,227^]^. It is vital to note that microglia to effectively extend their branches and ensure barrier integrity, they must be in a homeostatic P2RY12^+^ state. Inhibiting the function of P2RY12 can lead to negative secondary effects on the BBB^[^97^]^. Therefore, any condition that induces microglial reactivity and downregulation of P2RY12, including infection, neurodegeneration, or previous injury, impedes the barrier-sealing capacity following injury. These studies demonstrated that early purinergic receptor activity promotes neuroprotective immune responses after CNS injury. Responses mediated by purinergic receptors are critical mechanisms through which microglia maintain the integrity of glial limitans after mTBI. When these responses are inhibited, there is significant leakage of fluid from the subarachnoid space into brain tissue^[^81^]^.

### Interplays between extrinsic and intrinsic factors

Upon TBI, microglia are exposed to various external signals, including DAMPs, cytokines, chemokines, and growth factors, which collectively influence their functional responses. Furthermore, significant signaling pathways, including NF-κB and mitogen-activated protein kinase (MAPK), are activated in microglia, thereby regulating their inflammatory and reparative functions. The activation of inflammasomes in response to external signals further underscores their role in the inflammatory response after TBI. Understanding the interplay between these factors is vital for elucidating microglial functions in TBI and developing targeted therapeutic strategies aimed at improving outcomes in affected patients.

### Key signaling molecules associated with microglia in TBI

Microglia are vital for TBI. Multiple signaling pathways are involved in microglial polarization. Understanding how these pathways interact and their effects on microglial function is crucial for modulating microglial responses to TBI. In this context, we summarized the key classical signaling pathways involved in the microglial response to TBI.

**MAPK:** MAPKs represent a family of serine/threonine kinases that significantly contribute to signaling pathways activated by various extracellular stimuli, including TBI^[^228,229^]^. The mammalian MAPK family consists of c-Jun N-terminal kinases (JNK), extracellular signal-regulated kinases (ERK), and p38 MAPK^[^230^]^. The roles of ERK, JNK, and p38 MAPK, three members of the MAPK family, in neuronal survival and death appear to be complex and are modulated by various insults and conditions^[^231,232^]^. Previous studies have shown that blocking the activation of the MAPK signaling pathway may confer neuroprotective effects in patients with TBI^[^233,234^]^. One study found that p38α is an essential component of the intracellular signaling mechanisms that activate microglia in response to diffuse brain injury. This study also revealed that p38α has a major impact on the continued activation of microglia following injury, despite its acute inhibition of the peak cytokine response to diffuse brain injury^[^235^]^. These data suggest that the microglial response is localized specifically to the injured region in p38α KO mice, which could potentially be beneficial^[^235^]^. Another study demonstrated that the absence of p38α in microglia results in a pronounced decrease in cytokines and chemokines that promote inflammation across the tissue together with a considerable decrease in the infiltration of inflammatory monocytes. These findings indicate that microglial p38α contributes to the maintenance of a long-lasting and possibly neurotoxic inflammatory context in the brain after experiencing TBI^[^236^]^.

**PI3K/AKT:** The PI3K/AKT signaling pathway is integral to controlling organismal growth and a variety of cellular mechanisms, which makes it significant in many pathophysiological contexts^[^237^]^. An investigation has identified a strong relationship between dysfunction as part of the PI3K/AKT signaling pathway, phosphatase and tensin homolog (PTEN), which acts as the main negative regulator of AKT, chronic neuroinflammation, and microglial activation^[^238^]^. A previous study indicated that tetrahydrocurcumin may hold potential for treating TBI by promoting microglial M2 polarization and providing protection against nerve injury through the GSK3β/PTEN/PI3K/AKT signaling axis^[^239^]^. An enhancement of microglial exosomal miR-20b-5p caused by mild hypothermia can be transferred to injured neurons, promoting neurite growth and synaptic recovery after TBI by activating the PI3K/AKT pathway and reducing PTEN levels^[^240^]^. Histone deacetylases in microglia may represent a viable therapeutic strategy for TBI inhibition. This method indirectly supports oligodendrocyte survival by eliciting a GSK3β/PI3K/AKT-mediated change in microglial phenotype^[^241^]^.

**NF-κB:** NF-κB is a transcription factor formed from two subunits, p65 and p50. The NF-κB signaling pathway is normally retained in the cytoplasm by its inhibitor, IκB. Upon activation by stimuli, NF-κB rapidly dissociates from IκB-α and translocates to the nucleus, triggering a cascade of inflammatory responses. Several investigations have implicated the NF-κB signaling pathway in the inflammatory response associated with TBI^[^137,242,243^]^. NF-κB has a significant impact on the regulation of immune development, immune responses, and inflammation processes^[^244^]^. NF-κB activation promotes the transcription of genes responsible for producing pro-inflammatory cytokines, which in turn further activates NF-κB^[^245^]^. In microglia, the TLR-4/NF-κB signaling pathway serves as a classical transcriptional mechanism that regulates the expression of most M1-signature genes encoding pro-inflammatory cytokines^[^246^]^. Vascular endothelial growth inhibitors have the potential to diminish the intense inflammatory response after trauma and alleviate secondary brain injury by decreasing the TLR-4/NF-κB signaling cascade and the expression of associated inflammatory cytokines^[^247^]^. VX765 acts as a caspase-1 inhibitor that can lower pyroptosis and reduce activities associated with the HMGB1/TLR4/NF-κB pathway, thereby counteracting neurological damage post-TBI. This compound may exhibit a positive therapeutic effect in the context of TBI^[^248^]^. Abrocitinib, a selective inhibitor of Janus kinase 1 (JAK1), has demonstrated neuroprotective effects by diminishing the activity of the JAK1/STAT1/NF-κB pathway following TBI. This effect is mediated by a shift in microglial polarization from the pro-inflammatory M1 phenotype to the anti-inflammatory M2 phenotype^[^249^]^. ACT001 mitigated damage to the BBB and improved motor function following TBI by decreasing the microglial activation induced by trauma. This effect is associated with the AKT/NF-κB/NLRP3 signaling pathway^[^250^]^. In rats with ASDH, intracranial venous return disorders lead to alterations in cerebral blood flow and exacerbate neurological deficits and brain edema by compromising BBB integrity. This is attributed to the destruction of tight junction proteins in endothelial cells and upregulation of inflammatory factors, microglial activation, ADAM17 expression, and soluble TNF-α (solTNF-α) secretion. These factors subsequently increase the levels of IκB-α and NF-κB p65^[^251^]^. Research indicates that administering curcumin after injury may enhance patient outcomes by minimizing the acute activation of microglia/macrophages and decreasing neuronal apoptosis, which is thought to be mediated through the TLR4/MyD88/NF-κB signaling cascade in microglia in response to TBI^[^242^]^. A recent study indicated that electroacupuncture (EA) supports M2 polarization of microglia through blockade of the NF-κB/COX-2 signaling pathway, consequently providing neuroprotective benefits after TBI^[^252^]^. Hydroxychloroquine has been found to diminish the levels of pro-inflammatory cytokines, likely by inhibiting the TLR4/NF-κB signaling pathway, implying that HCQ may represent a valuable treatment candidate for TBI management^[^253^]^. Research has found that parthenolide decreases microglial activation and neuronal apoptosis by downregulating the STAT3/NF-κB signaling pathway, thereby conferring neuroprotective benefits in mouse models of TBI^[^254^]^. Following TBI, microglia release TNF-α, which amplifies neuroinflammation and oxidative stress due to the activation of downstream NF-κB/iNOS signaling, causing disruption of cerebral microcirculation mediated by pericytes^[^255^]^. In TBI rat models, 8-Methoxypsoralen modulates the PPARγ/NF-κB pathway, exerting neuroprotective and anti-inflammatory effects^[^256^]^.

**PPARγ:** PPARγ is a nuclear transcription factor expressed in microglia, monocytes, and macrophages^[^257,258^]^. PPARγ activation provides neuroprotection following TBI through anti-apoptotic, anti-inflammatory, and antioxidant mechanisms^[^259^]^. As a component of the nuclear hormone receptor superfamily, PPARγ is recognized for its potential anti-inflammatory properties in microglia and macrophages, as well as its neuroprotective role in TBI, largely due to its inhibition of phosphorylation and nuclear import of the NF-κB p65 subunit^[^259-261^]^. Pioglitazone activates PPARγ, which exerts neuroprotective effects and reduces inflammation following TBI^[^262^]^. The neuroprotective effects of phillyrin have been demonstrated in TBI models through activation of the signaling pathway involving STAT6 and PPARγ is pathways. Engaging PPARγ may serve as a therapeutic option for treating axonal injury following TBI by inducing microglial polarization to the M2 phenotype^[^263^]^. Bexarotene mitigates neurotoxicity in mice following TBI, partly through enhanced nuclear translocation and transcriptional activity via a PPARγ-dependent mechanism^[^264^]^.

Advances in single-cell RNA sequencing technology have enabled the study of complex and rare cell populations, revealing the gene-cell regulatory mechanisms involved in the pathogenesis of several diseases^[^265,266^]^. Future biological experiments utilizing specific Cre-loxP systems will facilitate the understanding of the precise roles of different microglial subpopulations in TBI.

### Inflammasomes

**Figure 2.**
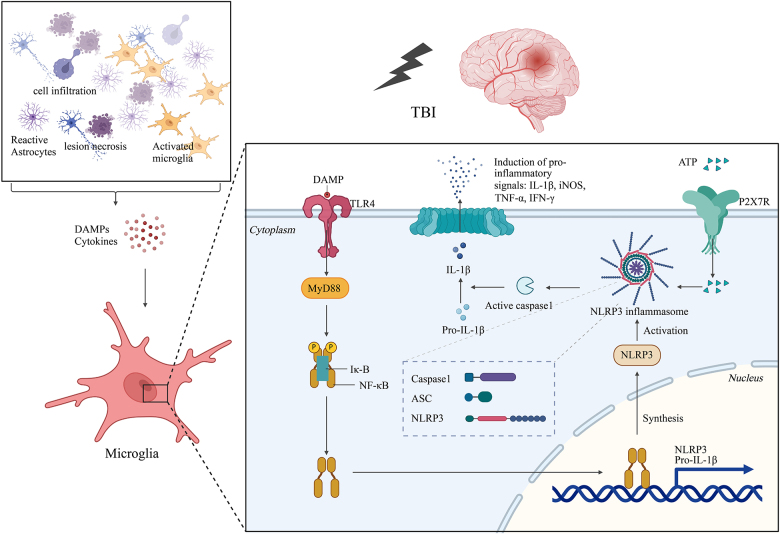
NLRP3 inflammasome activation following TBI. (1) Following an injury, neurovascular damage occurs immediately, resulting in the release of a significant amount of DAMPs. This release triggers the proliferation of microglia and the infiltration of peripheral immune cells. (2) DAMPs initiate TLR-mediated activation of NF-κB, subsequently leading to the expression of inflammasome genes. (3) ATP is released from damaged cells, activating the NLRP3 inflammasome via P2X7R. (4) The activation of the inflammasome occurs thereafter and involves the assembly of its constituent proteins, including NLRP3, ASC, and caspase-1, to form the NLRP3 inflammasome. (5) Ultimately, this complete inflammasome complex leads to the release of IL-1β. (Image created with BioRender.Com).

Inflammasomes consist of three essential components: a sensing element, procaspase-1 (serving as the effector molecule and an adaptor protein). The sensing element acts as a pattern recognition receptor (PRR) that identifies PAMPs and DAMPs. Alternatively, procaspase-1, a cysteine protease, is activated through the cleavage of pro-IL-1β and pro-IL-18 into their functional forms. Additionally, inflammasome activation leads to pyroptosis, a type of cell death that fosters inflammation^[^267^]^. Although not many studies are available, the data indicate that inflammasome proteins may act as possible biomarkers for evaluating the severity of TBI, as well as its outcomes and mechanisms of secondary injury^[^268^]^. In the CNS, PRRs are predominantly expressed by astrocytes, microglia, and macrophages. These cells are activated in the context of post-traumatic neuroinflammation^[^269-271^]^. Exploring the roles and mechanisms of inflammasomes in TBI could provide insights into novel therapeutic strategies that target neuroinflammation. PRRs are categorized into four primary functional and genetic classes: C-type lectins, membrane-associated receptors such as RIG-I-like receptors, TLRs, and NOD-like receptors (NLRs), which contain nucleotide-binding oligomerization domains^[^272^]^. NLRs are integral components of specific inflammasomes, notably NLRP3 and NLRP1 inflammasomes, which have recently undergone a comprehensive review^[^272^]^. Microglia serve as the primary source of inflammasome activation in the CNS. However, components of inflammasomes have been recognized in other resident cell types within the CNS, including neurons, astrocytes, oligodendrocytes, endothelial cells, perivascular macrophages^[^273^]^. The maturation of caspase-1 is driven by pro-caspase-1 in combination with an adaptor protein referred to as ASC (apoptotic apoptosis-speck-containing protein possessing a caspase activation and recruitment domain (CARD). In addition, caspase-1 contributes to the secretion of pro-inflammatory cytokines such as IL-1β and IL-18^[^162^]^. Most mechanistic insights into inflammasome function have been obtained from studies of rodent mononuclear phagocytic cells, particularly macrophages and microglia^[^274^]^. Nonetheless, certain features of inflammasome activation may vary between the macrophages and microglia. For example, conflicting findings in macrophages suggest that (LPS) priming of IL-1β expression in mouse microglia appears to necessitate the activation of caspase-8^[^275^]^. Several recent studies have reported an increase in inflammasome activity following TBI, primarily in activated microglia^[^276,277^]^. Molecules identified as activators of the inflammasome in microglia and macrophages during neurological diseases include A, prion proteins, and α-synuclein^[^278-280^]^. As DAMPs are frequently released upon neurological injury, ATP has also been proposed to function as a potential stimulus for inflammasome activation within the CNS^[^281-283^]^. Microglia are the primary contributors to the inflammatory activity in the CNS. The activation and formation of inflammasomes play crucial roles in regulating the inflammatory activity^[^284^]^. Inflammasomes are essential for addressing pathological inflammation, which results from the secretion of inflammatory cytokines and uptake of cell debris^[^284^]^. The impact of traumatic experiences on the CNS is a significant area of interest, particularly considering the parallels observed between disturbances in cellular homeostasis, activation of microglia and astrocytes, and inflammatory processes associated with the pathology of AD. Numerous studies have reported various phenomena, such as NLRP1, NLRP3, AIM2, ASC speck activity, the liberation of pro-inflammatory cytokines, pyroptosis, ongoing microglial activation, and damage to neurons reported in experimental studies following TBI^[^276,285-288^]^. Research has shown that the inflammasome is rapidly activated in microglia following the onset of intracerebral hemorrhage (ICH). Inflammasome components, including NLRP3, caspase-1 cleavage fragments, and IL-1β cleavage products, increase within 3 h of ICH^[^289^]^. Inhibiting inflammasome activation may protect microglia and macrophages from self-inflicted damage during phagocytosis, thereby maintaining them in an inflammatory-resolving phenotype that could facilitate hematoma clearance^[^290^]^. Following CNS trauma, microglia interact with astrocytes and neurons]. Astrocytes and microglia are essential for maintaining homeostasis in the CNS and modulating inflammatory cytokine expression, which is regulated by inflammasome assembly^[^291^]^. The processing and activation of IL-1β and IL-18 by a protein complex that forms an inflammasome can become hyperactive, resulting in the overproduction of these cytokines, which may contribute to pathologies associated with conditions such as AD, MS, and TBI^[^162^]^. The resolution of neuroinflammation is contingent on the severity of TBI. In instances of excessive and unresolved inflammatory responses, cytokines, including IL-1β, may stimulate the growth of glial cells in the area affected by TBI^[^162^]^. These findings highlight the critical interplay between inflammasome activation and neuroinflammatory responses after TBI. TBI is recognized as a contributing factor to the onset of AD as it increases inflammation and promotes the pathological onset of AD^[^284^]^. Specific features of neurodegenerative disorders, such as Aβ in AD and α-synuclein in Parkinson’s disease (PD), have been shown to facilitate inflammasome activation in microglia and macrophages. The subsequent activation of the inflammasome leads to the production of highly pro-inflammatory cytokines such as IL-1β and IL-18 by microglia and macrophages, thereby accelerating disease progression^[^290^]^. Traumatic injury leads to neuronal damage, exacerbated by the activity of Aβ and phospho-tau proteins, resulting in cell death and subsequent release of DAMPs, excitatory proteins, and ionic disturbances^[^292^]^. As a result, an ongoing inflammatory response occurs, characterized by heightened inflammasome activation that induces pyroptotic cell death, thus establishing a self-sustaining cycle of inflammation^[^284^]^. Murine studies have found that pro-inflammatory cytokines, particularly IL-1β, can disrupt the clearing activities of microglial cells, potentially hindering their ability to remove Aβ and other cellular debris^[^293^]^. Moreover, IL-18 has been shown to enhance β-site APP-cleaving enzyme activity and modify APP processing, potentially contributing to increased production of Aβ^[^294^]^. These insights into the interplay between neuroinflammation and neurodegenerative processes emphasize the need for precise treatment approaches. ASC particles are released into the surrounding extracellular space and are involved in increasing inflammasome activity. In the CNS, activated microglia take up ASC clusters, which subsequently secrete inflammasome-mediated cytokines. It has been shown that extracellular ASC specks are capable of propagating inflammation after being released by pyroptotic cells. Activated microglia subsequently take in these specks, which then produce inflammatory cytokines^[^295^]^. When microglia undergo further activation, cellular ASC levels increase, implying that microglia are vital participants in inflammasome processes following an initial injury^[^276,286^]^. A previous study revealed that applying an anti-ASC antibody to mice immediately post-TBI significantly reduced the volume of contusions compared to those that received vehicle treatment^[^287^]^. These findings underscore the critical role of ASC specks in the modulation of neuroinflammatory processes and highlight potential therapeutic targets for neurodegenerative diseases. The NLRP1 inflammasome was the first to undergo a comprehensive study and is also referred to by several other names, including NAC, DEFCAP, NALP1, CLR17.1, and CARD7^[^296^]^. NLRP1 is predominantly expressed in neurons responsible for movement in the cerebral cortex and spinal cord, with additional expression noted in microglia^[^272^]^. The NLRP1 inflammasome is essential for the early inflammatory response to TBI. Pharmacological inhibition of this complex has been shown to lead to histopathological improvements following TBI^[^297^]^. The clinical relevance of NLRP1 in TBI is underscored by elevated levels of NLRP1 expression observed in the CSF of TBI patients categorized as having adverse outcomes, whereas lower expression levels of these proteins were correlated with patients who exhibited favorable outcomes^[^268^]^. NLRP1 was detected in exosomes derived from the CSF of patients with spinal cord injuries and TBI^[^298^]^. Research findings suggest that the formation of a functional NLRP3 inflammasome is unique to microglia, rather than astrocytes, in the mouse brain. Furthermore, the study identified that NLRP3 inflammasome can be detected in neurons, microglia, and astrocytes within the rat cerebral cortex following TBI^[^271,299^]^. Ablation of Nlrp3 has been shown to protect the murine CNS from an age-related surge in the signaling pathways of two senescence-associated secretory cytokines that induce inflammation, such as IL-1β and IL-8^[^300^]^. The absence of NLRP3 leads to a reduction in neuroinflammation and Aβ aggregation, accompanied by advancements in locomotor skills, habituation, memory linked to the hippocampus, and synaptic adaptability in APP/PS1 mice. Furthermore, in APP/PS1 mice with NLRP3 and caspase-1 knockout, microglial activation resulted in enhanced phagocytosis of Aβ and promotion of an M2 phenotype known for its anti-inflammatory capabilities^[^293^]^. The effect of pioglitazone on NLRP3 modulation was examined in a mouse model of TBI by employing a weight drop mechanism. The results suggested that administration of pioglitazone led to less edema around the injury site, which was associated with decreased microglial and astrocyte activation and reduced NLRP3 inflammasome activity, as observed 14 days post-TBI^[^301^]^. Employing artesunate in a mouse model of CCI showed the ability to attenuate the inflammatory reaction induced by TBI, with a clear decline in the activation of the NLRP3 inflammasome and astrocytic and microglial cells. Furthermore, decreases in TNF-α, NF-κB, IL-1β, and iNOS expression levels have been documented 24 h post-TBI^[^302^]^. A separate investigation focused on dexmedetomidine (a selective α-2 adrenergic receptor agonist) and its effects in rats with cognitive dysfunction resulting from CCI. This treatment not only enhanced cognitive capabilities but also corresponded to a decrease in both microglial activation and NLRP3 inflammasome activity in the hippocampus^[^303^]^. NLRP3 expression is increased in aged microglia compared to young microglia^[^290^]^. Research indicates that the interaction between Aβ and ASC activates NLRP3, and that the formation of Aβ/ASC aggregates not only hinders Aβ removal but also leads to pyroptosis in microglial cells^[^304^]^. On the other hand, experiments using murine models have revealed that pTau activates the NLRP3 inflammasome in microglia, and tau pathology severity is reduced in mice genetically modified to lack ASC^[^305^]^. MCC950, categorized as a diarylsulfonyl urea derivative, is recognized for its exceptional selectivity and effectiveness in inhibiting the NLRP3 inflammasome The MCC950 treatment yielded promising early results, encompassing declines in NLRP3 and microglial activation, improvements in Aβ phagocytosis associated with AD, and reductions in the activity levels of caspase-1 and IL-1β following TBI^[^306^]^. These results underscore the therapeutic potential of targeting NLRP3 in neuroinflammatory conditions, paving the way for further research on anti-inflammatory treatments for neurodegenerative disorders. The roles of NLRP2, AIM2, and NLRC4 in microglia in the context of TBI remain relatively understudied and represent a promising avenue for future research (Fig. 2).

### Role of microglia in the neurogenesis of TBI

Neuroinflammation is a crucial component of the pathological microenvironment. The effects of posttraumatic neuroinflammation on adult neurogenesis can be either detrimental or supportive, depending on the specific cell types involved and their activation states^[^307^]^. While evidence suggests that microglia can promote neurogenesis, some research groups argue that these cells may also exert anti-neurogenic effects, highlighting the dual roles of microglia in adulthood^[^308-311^]^. In adult mammals, neurogenesis mainly occurs in two crucial areas of the brain: the subgranular zone (SGZ) of the dentate gyrus, located in the hippocampus, and the subventricular zone (SVZ), found in the lateral part of the ventricles. Notably, the SVZ is a component of the forebrain^[^312^]^. Ramified microglia found in the adult SGZ of the hippocampus serve a significant function within the neurogenic niche, enhancing the migration and differentiation of NSCs by releasing soluble factors that drive neurogenesis^[^313^]^. After brain injury, endogenous stimulation of adult neurogenesis is observed in the SVZ and SGZ, and areas devoid of neurogenic properties, such as the striatum and cerebral cortex, close to the site of injury^[^314,315^]^. LPS-induced inflammation, which markedly activates microglia, significantly decreases the number of recently developed neurons within the hippocampus of adult rats^[^309,316^]^. Moreover, microglia stimulated by LPS or their conditioned media have been demonstrated to decrease the survival of NSCs and inhibit their differentiation in vitro^[^317^]^. Research has repeatedly shown that many cytokines associated with inflammation, which are generated by M1 microglia and macrophages – including IFN-γ, IL-1β, TNF-α, and IL-6 – can considerably impede the process of neurogenesis^[^316,318-321^]^. IL-4 stimulation, conversely, causes microglia to become polarized into an M2-like phenotype, resulting in improved neurogenesis of NSCs in co-culture systems through elevation of IGF-1 levels, a vital cytokine. The correlation between higher IGF-1 levels and increased proliferation and neurogenesis in the hippocampus has been well established^[^322-325^]^. The involvement of microglial cells in adult neurogenesis is further supported by in vitro research, which demonstrates that NSCs can be cultured alongside microglia in conditioned media enriched with neurotrophic factors like basic fibroblast growth factor, brain-derived neurotrophic factor (BDNF), and nerve growth factor^[^311,313,326^]^. For example, the presence of microglia and their conditioned medium facilitates the in vitro production of neuroblasts from NSCs in the SVZ, which would have been irreversibly lost without this assistance^[^311,313^]^. In conclusion, the neuroprotective factors discussed previously, particularly serine protease 2 released from microglia, hold the promise to enhance the proliferation of neural precursors, aid in the movement of neuroblasts and enhance the functional assimilation of newly created neurons within the SGZ of the adult hippocampus^[^307,327^]^. Following TBI, the brain activates a natural response and/or plasticity mechanism that stimulates the growth of NSCs. However, chronic activation of proinflammatory pathways over an extended period can disrupt the proper integration of newborn neurons into their environmental niches. Consequently, there may be a need for additional support or interventions to boost endogenous neurogenesis and adequately restore the damaged brain tissue after TBI. Interventions aimed at increasing endogenous neurogenesis have been associated with enhanced recovery in adult rodents following TBI. In mice experiencing closed-head injury (CCI) that engage in a postponed voluntary exercise program with running wheels in their cages, significant decreases in the chronic activation of M1-like microglia have been detected following TBI. Furthermore, significant increases in the levels of IL-10, IGF-1, cAMP response CREB, and BDNF have been observed in the hippocampus, resulting in an elevated neurogenesis rate in the SGZ and enhanced cognitive abilities during the chronic phases^[^328^]^. Interactions between neurons and microglia play a critical role in determining the specific profiles of chemokines and cytokines released by microglia. For instance, neuron-derived fractalkine (CX3CL1; FKN) can stimulate the growth of new neurons in the adult hippocampus by acting on CX3CR1 receptors expressed on the microglia^[^329^]^. Pro-inflammatory microglia have been shown to secrete a collection of pro-inflammatory cytokines, such as TNF-α, IL-1β, and IL-6. This secretion has been demonstrated to induce death of the neurogenic NSC lineage while also reducing the complexity of immature newborn neurons^[^330^]^. Recent findings have indicated that the removal of microglia has a minimal impact on the outcomes of TBI. Conversely, the induction of a neuroprotective microglial phenotype has been shown to significantly enhance the survival of newborn neurons and mitigate cognitive impairment through IL-6 and IL-6 receptor signaling^[^175^]^. A promising strategy to address this clinical dilemma involves understanding the mechanisms by which neuroinflammation can act as both beneficial and detrimental factors, thereby harnessing its potential to promote neurogenesis. The proliferation of NSCs coincides with the expression of TLR4, suggesting an intricate link between neurogenesis and inflammation. This relationship has spurred interest in the regulation of inflammation following TBI, particularly regarding the significance of NSCs in overseeing of NLRP3 and IL-1β, potentially reducing the neurotoxic cascade elicited by microglia^[^331,332^]^. Following transplantation, NSCs/neural precursor cells have been observed to alter the microglial phenotype and ameliorate the inflammatory response. These observations suggest that extracellular vesicles (EVs)obtained from NSCs may also exhibit anti-apoptotic and anti-inflammatory functions^[^333-335^]^. Microglia communicate with the surrounding tissue and different neighboring cell types found in the brain, such as astrocytes and neurons, to conduct immune surveillance and maintain NSC homeostasis^[^150,336,337^]^. In an inflammatory context, the alternatively activated phenotype demonstrates sustained and supported neurogenesis. Conversely, the activated phenotype is characterized by diminished capacity for neurogenesis in a pro-inflammatory state. The exceptional phagocytic abilities of quiescent microglia provide significant support for hippocampal neurogenesis^[^213,338^]^. The exceptional phagocytic ability of quiescent microglia provides significant support for hippocampal neurogenesis. This support is achieved through the clearance of apoptotic neurons and facilitation of neuronal integration within the hippocampal circuitry^[^339^]^. Research conducted by Walton *et al* provided further evidence that resting microglia play a regulatory role in hippocampal neurogenesis. Their findings demonstrated that resting microglia release factors that influence neuronal differentiation^[^313^]^. The proinflammatory cytokines involved in this process include TNF-α, IL-6, and IL-1β. These cytokines have been shown to exert a negative regulatory effect on hippocampal neurogenesis by reducing the increase in NSCs and the formation of novel neurons This results in a shift toward gliogenesis, accompanied by a corresponding reduction in neurogenesis^[^213,338,340,341^]^. Additionally, after intraperitoneal administration of LPS to rats, a reduction in neurogenesis and a pronounced increase in microglial activation were recorded, with no influence on NSC proliferation^[^316^]^. Typically, microglia are predisposed to favor neurogenesis instead of gliogenesis. However, stressors, such as aging or injury, can activate microglia, triggering the production of pro-inflammatory cytokines that promote gliogenesis, often to the detriment of neurogenesis. Subsequently, microglia assume an immunomodulatory role marked by the release of anti-inflammatory cytokines, thereby renewing their emphasis on neurogenesis^[^342^]^. Therefore, quiescent microglia support hippocampal neurogenesis by clearing apoptotic neurons and releasing the factors that promote neuronal differentiation. However, when activated by stressors, these microglia release pro-inflammatory cytokines that suppress neurogenesis and encourage gliogenesis, although they can shift to an immunomodulatory state that favors neurogenesis.

### Role of microglia in the angiogenesis of TBI

Angiogenesis is a crucial process in the restoration of injured brain tissue and plays a significant role in functional recovery following TBI^[^343^]^. Research indicates that the application of drugs designed to enhance angiogenesis can lead to better functional recovery after experimental TBI^[^344,345^]^. In the acute phase of injury, inflammatory monocytes assist in the onset of cerebral edema but further stimulate the growth of new blood vessels. Depletion or inhibition of microglial activity, as well as the purinergic response, during cerebral vascular injury (CVI) triggers considerable vascular leakage and collateral damage to the brain parenchyma^[^94^]^. After vascular injury, microglia significantly contribute to the development of a quasi-barrier. Deficiencies in this process can lead to secondary brain damage^[^87^]^. In a brain with sustained injury, remodeling associated with angiogenesis occurs in the ischemic penumbra shortly after the initial ischemic damage^[^346^]^. The observation of microglia and macrophages around angiogenic vessels suggests that these cells are likely essential for the angiogenic response to cerebral ischemia^[^347,348^]^. Microglial activation is known to govern the proliferation of brain endothelial cells, an early phase of cerebral angiogenesis, by modulating the interplay between TNF-α, which promotes inflammation, and TGF-β, which inhibits inflammation^[^349^]^. Studies have indicated that treating brain endothelial cells with conditioned media from M2 microglia polarized with metformin promotes angiogenesis in vitro^[^350^]^. M2 macrophage-mediated angiogenesis is facilitated by the production of growth factors and factors that support angiogenesis, including vascular endothelial growth factor (VEGF) and fibroblast growth factor 2 Additionally, the release of matrix metalloproteinase-9 propeptide and matrix metalloproteinase-2 contributes to this process^[^73,351,352^]^. It has also been shown that a group of microglia linked to gliomas in humans can secrete VEGF-A, leading to enhanced angiogenesis in rodent brain tumors^[^353,354^]^. VEGF-A stimulates endothelial cell proliferation and migration and serves as a potent inducer of angiogenesis. In patients with acute ischemic stroke and stroke-induced rodents, activated non-phagocytic microglia have been found to express VEGF in areas surrounding damaged brain tissue^[^355-357^]^. Research has revealed that the mechanism of angiogenesis after cerebrovascular damage depends on microglial clusters that express the proangiogenic factor VEGF-A. A defined population of repair-associated microglia (RAM), marked by CD45^int^ CX3CR1^+^ P2RY12^+^ CD24^+^ profiles, was found to aggregate around injured blood vessels while producing VEGF-A. In microglia-deficient mice, angiogenesis-related genes did not show increased expression, and new blood vessel development failed to occur following CVI. Additionally, invading monocytes facilitate pro-angiogenic functions in microglia, as RAM formation is impaired when the influx of immune cells from the blood is impeded^[^94^]^. Overexpression of pro-angiogenic factors, specifically VEGF and FGF, following experimental it has been shown to provide neuroprotective benefits, leading to increased post-traumatic angiogenesis and neurogenesis. Together, these processes contribute to enhanced functional recovery^[^358-360^]^. Research findings from both in vitro and in vivo studies suggest that M2-polarized microglia and macrophages play a key role in angiogenesis and vascular restoration following traumatic injury (Fig. 1).

### Energy metabolism

As the resident macrophages of the CNS, microglia perform a variety of essential functions, including monitoring pathogens, facilitating tissue restoration, pruning synapses, and supporting neuroplasticity, all of which are crucial for learning^[^112,361^]^. The high-energy demands of the immune system and its complex functions necessitate metabolism of glucose, amino acids, lipids, and ketone bodies. Considering the gradual advancement of secondary brain injury, studying regional variations in microglial metabolism may provide novel insights into immunometabolic imbalances and the widespread neurotoxicity and damage that occur after TBI^[^362^]^.

Moreover, these metabolic pathways generate intermediate metabolites that act as biosynthetic precursors in reactions governing shifts between various inflammatory states^[^363-366^]^. The utilization rates of substrates and catabolic pathways involved are linked to the phenotypic states of microglia^[^367,368^]^. The term immunometabolism refers to shifts in the metabolic pathways of cells that occur during immune response activation^[^369^]^. Deficiencies in immunometabolic responses have been found to contribute to the emergence of harmful pro-inflammatory microglial phenotypes related to neurodegeneration^[^370,371^]^. Following TBI, several crucial enzymes that display persistent dysfunction in microglia rely on intermediates from energy metabolic pathways, including the tricarboxylic acid cycle, glycolysis, and glutaminolysis. These pathways provide substrates for processes associated with pro-inflammatory microglial activation, such as ROS production, illustrating the important role of metabolic remodeling in sustaining chronic microglial activation and neuroinflammation^[^372^]^. These data suggest that microglial plasticity, particularly in terms of a pro-inflammatory activation state, is shaped by glucose-dependent metabolic pathways reliant on glucose. Microglia in a pro-inflammatory state demonstrate increased mRNA levels of *GLUT-1* and hexokinase-2 (*HK-2*)^[^373^]^. By inhibiting HK activity with agents such as 2-deoxyglucose, 3-bromopyruvic acid, siRNA directed against HK-2, or GLUT-1 siRNA to block glucose uptake, LPS-triggered pro-inflammatory activation in microglia is notably reduced^[^370,374^]^. In pro-inflammatory microglial activation, heightened glucose uptake seems to be largely facilitated by NADPH production mediated by the pentose phosphate pathway instead of being driven by the energy produced via glycolysis^[^371^]^. A comparison of lipid composition in the entire brain and microglia revealed notable differences. Microglia show increased concentrations of polyunsaturated fatty acids (PUFAs), especially eicosapentaenoic acid and docosahexaenoic acid (DHA)^[^375^]^. PUFAs play a role in modulating neuroimmune responses, and changes in omega-3 (ω-3) and omega-6 (ω-6) PUFAs affect the transition between proinflammatory and anti-inflammatory microglial phenotypes^[^376^]^. A further preclinical study found that lipidomic modifications were still observable 7 days post-injury, along with heightened levels of unsaturated fatty acids (FAs) (18:0) and PUFAs, including arachidonic acid and DHA^[^377^]^. Furthermore, providing omega-3 (ω-3) PUFA supplements to injured WT mice resulted in a reduction of microglial and macrophage activation and facilitated better long-term neurological recovery, reaching levels similar to those in fat1^+^ transgenic mice after injury^[^378^]^. Although the exact relationship between cholesterol metabolism and neuroimmune responses after trauma is still not fully elucidated, in vitro research suggests that a reduction in cholesterol transport within microglia, accomplished by knocking out the ATP-binding cassette protein ABCA1, intensifies pro-inflammatory microglial activation^[^379^]^. Another study using lipidomic analyses found that following TBI, there is an acute rise in cholesterol esters in microglia, which persists for a week after the injury occurs in both the injured cortex and hippocampus^[^380^]^.

## The crosstalk between microglia with other cell types in TBI

Imbalances in brain homeostasis can cause modifications in cellular interactions, which are implicated in the etiology of numerous neurological diseases such as TBI, SCI, AD, and PD^[^381^]^. Microglia interact with various brain cells, play an essential role in maintaining brain homeostasis under healthy conditions, and react to warning signs in the context of neurological damage or illness^[^382^]^. Although the primary influence of microglial cells on injury or illness outcomes is evident, their actions are part of a broader network. The activities and reactions of microglia in response to cerebral injury occur through their connections with neurons and other types of glial cells^[^383,384^]^. It has been hypothesized that microglia act as intrinsic immune cells within the brain, initiating an inflammatory response in response to injury. This response leads to the activation of astrocytes along with endothelial cells. Afterwards, Immune cells present in the peripheral system, including neutrophils, monocytes, and lymphocytes, are recruited to the injured area where they synthesize multiple inflammatory factors, including cytokines and chemokines. These mediators facilitate robust and intricate interactions between cells, thereby contributing to post-TBI inflammation.

### Crosstalk between microglia and neurons in TBI

Immune suppression in the CNS is a crucial factor in maintaining the normal physiological condition of microglial cells. In a healthy neural environment, neurons generate various immunoregulatory proteins that serve as “inhibitory signals,” interacting with specific receptors on microglia to prevent their activation^[^385^]^. Microglia are essential for the digestion of cellular debris following brain injury because of their phagocytic capabilities. In addition, they synthesize important neurotrophic factors and anti-inflammatory cytokines that are critical for preventing ongoing neuronal damage and encouraging the healing of tissue integrity. The transition to a disinhibited and highly reactive microglial activation state causes the overproduction of pro-inflammatory and cytotoxic substances, which results in neuronal dysfunction and ultimately leads to cell death^[^107^]^. In addition, neurons, astrocytes, and microglia emit soluble signals into the surrounding environment, such as neurotrophins, anti-inflammatory cytokines, and prostaglandins, which regulate immune responses. This function keeps microglia in a vigorous state^[^336^]^. Exposure of cultured microglia to conditioned medium from ischemic neurons induced an M1-like phenotype, indicated by elevated levels of pro-inflammatory mediators, such as TNF-α and nitric oxide, leading to a reduction in the phagocytic function of microglia. This implies that factors released from injured neurons are essential in helping microglia transition from an M2 to an M1 phenotype.

In situations of injury or illness, signals arising from neuronal damage can help explain microglial activation. It is widely accepted that activated microglia are involved in both neuroprotective and neuroinflammatory processes. Persistent microglial activation can result in a chronic neuroinflammatory state that threatens neuronal integrity and disrupts communication pathways between neurons and microglia. They interact through a bidirectional process that includes the release of EVs, facilitating the exchange of a range of molecules such as intracellular signaling components and second messengers. Specifically, mobile EVs released by activated microglia help to transmit pro-inflammatory cytokines that promote neuroinflammation in distant brain regions. Dendritic synapses typically act as sites of direct contact-dependent interactions between neurons and microglia. Furthermore, studies have shown that microglia directly interact with axons^[^386^]^. Microglia can interact with presynaptic and postsynaptic neurons at the synapse and establish connections with both inhibitory and excitatory synapses^[^387,388^]^. In TBI, DAMPs provoke an immune response from glial cells, which in turn contributes to neuroinflammation. Once activated, glial cells, particularly microglia, produce cytokines and chemokines in response to this initial signal. This preliminary paracrine communication between damaged neurons and microglia activates the inflammatory cascade linked to brain injury, and activated microglia release early mediators of neuroinflammation, identifying pro-inflammatory substances, such as TNF-α, IL-6, and IL-1β. After their release, these inflammatory substances stimulate ROS production in neurons, possibly leading to axonal damage. In addition, microglia react to the produced ROS by amplifying the release of the same pro-inflammatory cytokines (TNF-α, IL-6, and IL-1β), forming a feedback loop that aggravates inflammation during the injury phase^[^66,389-391^]^.

In the CNS, microglia display significantly high levels of chemokine receptor 1 (CX3CR1). The unique ligand of this receptor, fractalkine (CX3CL1), is synthesized by neuronal cells. CX3CL1 is known for its role in triggering microglial activation and enhancing the release of pro-inflammatory mediators^[^392^]^. In physiological states, the CX3CL1-CX3CR1 signaling pathway enhances the dialog between healthy neurons and microglia, permitting microglia to track neuronal health while curbing their own activation. However, in pathological states, such as TBI, communication from injured neurons cannot be maintained, preventing microglia from being kept in an inhibited state^[^393^]^. Evidence suggests that, while microglia-mediated fractalkine receptor signaling is associated with early toxicity, it is also vital for later protective effects caused by TBI^[^394^]^. Neurons express CX3CL1 to draw into adjacent microglia through a “find me” mechanism, which affects the maturation of synapses; however, the specific details of this process still need to be elucidated^[^395^]^. In the aftermath of an event that injures the brain parenchyma, there is a surge in glutamate release, which activates NMDA receptors found on the surface of neurons. This activation leads to the secretion of soluble CX3CL1, which triggers microglia to release IL-1β, thus increasing the excitability of adjacent neurons. Moreover, this triggers localized ATP release at targeted dendritic spots, attracting the processes of nearby microglia through activation of P2Y12, leading them to gather at the release location^[^396^]^. The results showed that *CX3CR1^−/−^* mice exhibited milder sensorimotor issues and less neuronal damage 4 days after TBI than their WT counterparts, who also sustained injuries. This finding indicates that fractalkine signaling may contribute to neurotoxicity during the early post-TBI phase^[^394^]^. Conversely, at five weeks post-TBI, compared to WT mice, *CX3CR1^−/−^* mice experienced heightened neuronal cell death and sensorimotor dysfunction suggesting that fractalkine signaling may offer neuroprotective effects during this later period following TBI^[^394^]^. Researchers found that microglia showed M2 markers, including Ym1, CD206, and TGF-β, in the acute injury phase, whereas they primarily displayed M1 markers, such as CD68, during the chronic phase. This transition in phenotypic expression is linked to the differing effects of CX3CR1 deletion after TBI^[^397^]^.

CCL21 acts as a chemokine that is emitted by damaged neurons, which activates and recruits microglia via its receptor CXCR3. Furthermore, the secretion of CCL21 by neurons increases the level of P2X4 receptor expression in microglia, which is crucial for the progression of neuropathic pain following nerve injury^[^398^]^. In cases of TBI, microglia converge in areas of neuronal injury, a process driven by ATP^[^81^]^. ATP is released from astrocytes and neurons in the area of injury, resulting in a localized concentration that aids in the convergence of microglia. ATP interacts with P2Y receptors on microglia, stimulating the extension of a single process toward the injury site, which leads to the conversion of ramified microglia into an active form with elongated processes. This role of ATP was substantiated by the transcranial administration of inhibitors targeting purinergic receptors (P2RY12 or P2RX4) prior to compression injury, effectively preventing the occurrence of this phenomenon from occurring^[^81^]^. In the context of injury, phagocytosis is a vital interaction between the microglia and neurons. Upon activation, microglia phagocytose and dispose of axonal and myelin debris, caused by Wallerian degeneration of the distal axonal segment. This mechanism involves various receptors, including TLRs, TREM2-expressed triggering receptor, complement receptors 3 and 4, macrosialin, and purinergic receptor P2RY6, which assist in the engulfment of myelin^[^399^]^. TREM2, found in microglia, stimulates the activation of these cells and facilitates phagocytosis, thereby helping clear apoptotic neurons. It collaborates with DAP12 to create a receptor-adaptor complex that changes the phagocytic properties of microglia^[^400^]^. The release of matrix metalloproteinase 3 from apoptotic neurons activates microglia, resulting in the production of proinflammatory cytokines, including TNF-α, IL-6, and IL-1β. Microglia activation is also mediated by ERK phosphorylation^[^401^]^. In the context of disease, CCL2 expressed by neurons and astrocytes activates and recruits microglial cells through its receptor, CCR2. This CCL2-CCR2 signaling cascade is significant because it triggers the phosphorylation of STAT3, which afterwards increases IL-1β production and is linked to neuronal cell death^[^402,403^]^. Following neuronal injury, excessive glutamate, a key excitatory neurotransmitter in the CNS, can cause further neuronal cell death. Glutamate activates the mGlu2 receptor in microglia, resulting in the production of pro-inflammatory TNF-α, which exacerbates neurotoxicity. Furthermore, glutamate releases Fas ligand, which activates Fas receptor and caspase-3, ultimately leading to neuronal cell death via apoptosis^[^404,405^]^. Understanding how communication mechanisms operate and the role of their dysfunction in neurological diseases is vital for creating innovative therapeutic interventions that specifically target abnormalities in communication between microglia and neurons. This knowledge is expected to facilitate the development of targeted therapies to address these specific communication disruptions (Fig. 3).

### Crosstalk between microglia and astrocytes in TBI

The BBB comprises a range of structural components and cellular structures, including pericytes, vascular endothelial cells, glial limitans, the perivasculitis (astrocytes), and microglia. However, the integrity of the BBB is frequently compromised in various immunopathological conditions, necessitating rapid restoration to re-establish homeostasis^[^73,94^]^. The unique endothelial cells that form the vasculature of the CNS are fundamental units of the BBB. These endothelial cells differ from those that create blood vessels located in the peripheral nervous system, including those found in the dura mater. Moreover, they are surrounded by a basement membrane and wrapped by pericytes. Glial limitans consist of astrocytic end-feet and delineate the perivascular space along with the basement membrane^[^406^]^. Astrocytes are crucial for preserving homeostasis in the CNS by forming an important barrier between the blood, CSF, and meninges. The occurrence of glial scars after injury is a protective strategy that reduces damage and inflammation in the affected area. This mechanism, called reactive astrogliosis, represents a neuroprotective response that can occur independently of cell death^[^81^]^. Astrocyte neighboring lesions exhibit increased reactivity, higher levels of glial fibrillary acidic protein (GFAP), and enhanced production of cytokines and chemokines. This heightened activity contributes to the activation and recruitment of the resident microglia and peripheral immune cells. In mice, ATP detection through the purinergic receptor P2RY12 prompts the rapid extension of microglial processes towards the glial limitans superficialis, where they help seal the spaces between astrocytes. Following the death of glial-limited astrocytes after mTBI, microglia have been found to undergo further activation in response to P2RY6 signaling. This transformation results in a jellyfish-like shape, enabling microglia to fill the gaps left by the death of nearby astrocytes and phagocytose cellular debris^[^81^]^. The two responses dependent on purinergic receptors are important ways in which microglia help maintain glial integrity of glial limitans after mTBI. Inhibiting these processes leads to marked fluid leakage from the subarachnoid space into the brain’s parenchyma^[^81^]^. It is vital to point out that microglia must remain in a stable P2RY12^+^ state to promote the projection of their processes and sealing of barriers. Any situation that leads to microglial reactivity and subsequent downregulation of P2RY12, such as infection, neurodegeneration, or prior injuries, interferes with their ability to seal off barriers following injury^[^407-410^]^. Additionally, research has demonstrated that microglia promote astrocyte-mediated toxicity during episodes of inflammation. This mechanism depends on the NF-κB signaling cascade within microglia and is associated with the specific circumstances involved^[^411^]^. Thus, although microglia first exhibit a supportive role for astrocytes, compensating for BBB deficits due to astrocyte death, they later have a harmful effect on the same cells (Fig. 3).

### Crosstalk between microglia and macrophages in TBI

TBI triggers rapid and localized immune responses in both humans and animals. This inflammatory process is marked by complex interactions between cells and involves both the intracerebral and peripheral immune populations. Although research has typically concluded that myelomonocytic cells can have either a beneficial or harmful effect on outcomes following CNS injury, it is crucial to appreciate that these cells can play different roles in the same injury context, depending on the timing, spatial factors, and their specific functions^[^135,412,413^]^. Research has demonstrated that both microglial cells and brain macrophages display numerous shared protein markers such as CD11b and CX3CR1. In accordance with local chemokine signals, such as those produced by CCL2, CXCL10, and CCL5, monocytes are summed up in the affected area of the brain. Once these cells have arrived, they differentiate into macrophages. A recent study revealed two subpopulations of monocytes differentiated by their expression of chemokine receptors CCR2 and CX3CR1. CCR2^+^ cells are categorized as “inflammatory” monocytes (CD11b^+^ CD45^hi^ CCR2^+^ Ly6C^hi^), while CX3CR1^+^ cells are referred to as “patrolling” monocytes (CD11b^+^ CD45^hi^ CX3CR1^+^)^[^414^]^. Inflammatory monocytes are selectively directed to the site of TBI, accounting for the majority of lesion three days post-injury^[^415^]^. A review of the available literature on cerebrovascular restoration during the 7 days following CVI found that myelomonocytic cells, particularly those that are CCR2^+^, play a vital role in angiogenesis. This effect is mediated by granting microglia angiogenic properties, which include the ability to generate VEGF-A^[^416^]^. Inhibiting the recruitment of monocytes during CNS recovery limits the formation of RAMs and negatively affects angiogenesis^[^416^]^. Therefore, myelomonocytic cells can initially harm blood vessels after CVI but subsequently assist in their restoration. These results have important implications for the timing of therapies aimed at peripheral cells, such as the blockade of LFA1/VLA4. Besides supporting parenchymal angiogenesis and enhancing interactions among microglia, studies have demonstrated that peripheral monocytes play a role in restoring damaged blood vessels in the meninges after mTBI^[^73^]^. Importantly, macrophage infiltration interferes with microglia-mediated phagocytosis and the subsequent inflammatory responses. Infiltrating macrophages exert a suppressive effect on microglial activation by diminishing the expression and ingestion of inflammatory molecules, thereby preventing chronic microglia-driven inflammation in the CNS. Targeting these two cell populations requires a careful approach and deep understanding of their intricate and specific roles in injury and disease. According to research, peripheral macrophages influence the NF-κB signaling pathway in microglia, subsequently lowering the levels of pro-inflammatory mediators, such as TNF, which could help prevent toxicity mediated by astrocytes^[^411,417^]^. Consequently, TBI initiates a localized immune response that features complex interactions between intracerebral and peripheral immune cells, with the impact of myelomonocytic cells varying according to the injury scenario. While inflammatory monocytes may initially cause damage and promote angiogenesis, infiltrating macrophages can inhibit detrimental microglial activation and inflammation. This indicates the need for a careful therapeutic approach when targeting these distinct cell subsets (Fig. 3).

### Crosstalk between microglia and oligodendrocytes in TBI

Myelinating oligodendrocytes exhibit marked susceptibility to ischemic or traumatic damage^[^418,419^]^, with oligodendrocyte depletion representing a pivotal determinant of post-injury demyelination^[^420^]^. Oligodendrocytes exhibit exquisitely vulnerable to diverse pathological stimuli – notably neuroinflammation, oxidative stress^[^421^]^, and excitotoxic cascades^[^422,423^]^ – each precipitating oligodendroglia apoptosis and subsequent demyelination following cerebral ischemia or traumatic injury. Microglia exhibit a dichotomous regulatory influence on post-traumatic WM pathology. In their deleterious capacity, microglia liberate ROS and pro-inflammatory mediators including TNF-α, exerting cytotoxic effects on adjacent oligodendroglia populations. Furthermore, activated microglial phenotypes demonstrate glutamatergic hyperactivity^[^424^]^, potentiating excitotoxic insults to both mature oligodendrocytes and their progenitor pools within compromised CNS territories^[^425^]^. Conversely, reactive microglia may mediate the resolution of neuroinflammation, facilitate the phagocytic clearance of cellular debris, and secrete neuroprotective mediators that mitigate oligodendroglia damage or demyelination^[^336^]^. Pharmacological or genetic inhibition of NHE1 protein activity enhanced post-traumatic neurological recovery through attenuating microglial pro-inflammatory polarization, curbing neurodegenerative cascades, and promoting oligodendroglia regeneration during WM restitution^[^426^]^. TREM2 exhibited peak expression at 3 days post-TBI, with principal localization to WM-resident microglial populations. Genetic ablation of TREM2 exacerbated TBI-induced neurological deficits, a phenomenon mechanistically linked to aggravated WM pathology, diminished oligodendrocyte precursor cell renewal, and compromised microglial phagocytic capacity^[^427^]^. Pharmacological blockade of IL-1β – a pivotal mediator of post-traumatic neuroinflammation – augments oligodendrocyte survival and stimulates oligodendrocyte precursor cell proliferative activity through modulating microglial/macrophage immunophenotypic responses to traumatic CNS insults^[^428^]^. The dual role of microglia in post-traumatic WM pathology highlights their pivotal regulatory function in CNS injury. Their ability to both exacerbates oligodendroglia damage and facilitate recovery under specific conditions underscores the dynamic balance that governs WM recovery. Thus, targeting key molecules such as NHE1, TREM2, and IL-1β not only holds potential for mitigating injury progression but also represents a promising therapeutic strategy for promoting tissue recovery and functional restoration.

## Application of scRNA-seq in studying microglia

**Figure 4.**
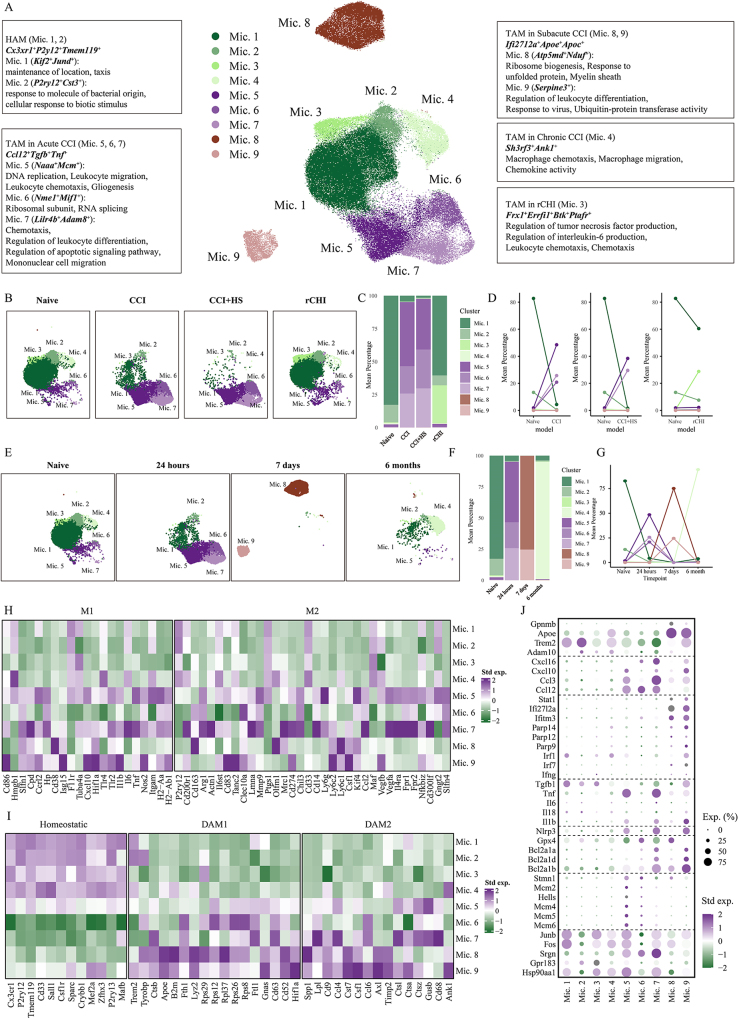
Microglia subpopulation diversity in TBI. (A) Microglial subpopulations. (B-D) Distinct TAM signatures across different TBI models. (E-G) Transition of TAMs from acute neuroinflammation to chronic maladaptive plasticity over time in CCI. (H) Expression of M1 and M2 marker genes in the nine identified microglial subpopulations. (I) Expression of homeostatic markers, DAM1, and DAM2 in the nine identified microglial subpopulations. (J) Expression of TBI-related factors in the nine identified microglial subpopulation.

The single-cell RNA sequencing (scRNA-seq) dataset utilized in this study was derived from a publicly available resource established by Ruchira *et al*^[^429^]^, which provides comprehensive transcriptional profiles of microglia across multiple TBI paradigms. Through comprehensive scRNA-seq analysis, we delineated two principal axes of microglial heterogeneity in TBI: 1) Model-driven diversity: Cross-injury comparisons among clinically relevant TBI models – including controlled cortical impact (CCI, modeling focal cerebral contusion), CCI with hemorrhagic shock (CCI + HS, modeling polytrauma with concurrent brain injury and systemic hemorrhage) and repetitive closed-head injury (rCHI, modeling sports-related concussions), – revealed distinct TAM transcriptional signatures at 24 h post-TBI; 2) Time-dependent evolution: Longitudinal analysis of CCI-exclusive cohorts (24 h, 7 days and 6 months post-injury) identified temporally stratified TAM activation states, transitioning from acute neuroinflammation to chronic maladaptive plasticity. This temporal resolution highlights the dynamic progression of microglial responses over the course of TBI recovery and pathology. Unsupervised clustering based on subtype-specific molecular signatures resolved microglial populations into nine transcriptomically compartments (Mic. 1-9) (Fig. 4A). Among these, Mic. 1 and Mic. 2 were characterized by enriched expression of homeostatic markers, leading to their designation as Homeostasis-associated Microglia (HAM) (Fig. 4A). At 24 h post-CCI, HAM populations (Mic. 1, Mic. 2) were significantly diminished, while Mic. 5, Mic. 6, and Mic. 7 exhibited pronounced expansion. This pattern was consistently observed in both CCI and CCI + HS groups, suggesting a shared response to focal injury and systemic trauma. Based on these dynamic transcriptional profiles and injury-specific expansion, we designated Mic. 5/6/7 as CCI-specific TAM (TAM-C). In contrast, the rCHI model induced selective enrichment of Mic. 3 at 24 h post-injury. This population displayed unique activation patterns, distinct from those oberved in CCI and CCI + HS models and was consequently defined as rCHI-specific TAM (TAM-R) (Fig. 4B-D).

To elucidate the functional roles of microglial subpopulations, we conducted differential genes using bioinformatics tools, integrating pathway enrichment and functional annotation to identify key biological processes and signaling pathways associated with each cluster. The TAM-C subclusters displayed a core pro-inflammatory axis, characterized by pan-subtype upregulation of inflammatory mediators such as *Ccl12, Tgfb*, and *Tnf*. These findings suggest a coordinated inflammatory response across TAM-C populations. Mic. 5 (*Naaa^+^Mcm^+^*): This subcluster was enriched in pathways related to DNA replication and leukocyte chemotaxis, indicative of proliferative expansion and immunoregulatory recruitment. These features align with its role in amplifying glial reactivity, particularly gliogenesis, during acute secondary neurodegeneration. Mic. 6 (*Nme1^+^Mif^+^*): This subcluster exhibited activation of ribosomal biogenesis and RNA splicing, reflecting metabolic adaptation to sustain protein synthesis. Additionally, *Nme1*-driven nucleotide metabolism may balance energy demands during reparative gliosis. Mic. 7 (*Lilr4b^+^Adam8^+^*): Dominated by chemotactic signalling and pathway regulating leukocyte differentiation and apoptosis, this subcluster appears to play dual roles in myeloid cell recruitment and phagocytic clearance. Collectively, these TAM-C subclusters orchestrate spatially resolved crosstalk between inflammation, proliferation, chemotaxis, and metabolic reprogramming, highlighting their hierarchical roles in TBI pathogenesis. The TAM-R (Mic. 3) subcluster showed marked upregulation of *Frx1, Errfi1, Btk*, and *Ptafr*, alongside pathway enrichment in TNF production, IL-6 signaling, and leukocyte chemotaxis. This transcriptional profile suggests a dual role in pro-inflammatory amplification (mediated by *Btk*-dependent NF-κB activation, driving TNF and IL-6 cascades^[^430^]^) and chemotactic recruitment (facilitated by *Ptafr*-dependent lipid signaling^[^431^]^, promoting myeloid infiltration). The co-expression of *Frx1* and *Errfi1* further implies metabolic adaptation to sustain redox homeostasis^[^432^]^ and meet energy demands^[^433^]^ during chronic neuroinflammatory stress (Fig. 4A).

At 7 days post-CCI, subacute TAM exhibited marked upregulation of *Ifi2712a, Apoe*, and *Apoc*, with transcriptional divergence delineating two injury-responsive subsets (Mic. 8, Mic. 9). Mic. 8 (*Atp5md^+^Nduf^+^*): This subset demonstrated enrichment in pathways related to Ribosome Biogenesis, Response to Unfolded Protein, and Myelin Sheath Remodeling, indicative of heightened protein synthesis capacity and metabolic adaptation to endoplasmic reticulum stress. The upregulation of *Nduf* genes further suggests a role in mitochondrial energy metabolism^[^434^]^. Mic. 9 (*Serpine3^+^*): Dominated by pathways such as Regulation of Leukocyte Differentiation, Response to Virus, and Ubiquitin-Protein Transferase Activity, this subset appears to play critical roles in immunomodulation and photostatic regulation through ubiquitin-mediated degradation. Collectively, these spatially compartmentalized phenotypes orchestrate proliferative renewal, inflammatory resolution, and myelin repair, highlighting their hierarchical contributions to secondary neurodegeneration and chronic neuroinflammatory sequelae. At 6 months post-CCI, Mic. 4, representing chronic-phase TAM, demonstrated marked upregulation of *Sh3rf3* and *Ank1*, alongside pathway enrichment in macrophage chemotaxis, migratory regulation, and chemokine activity. These mechanisms collectively reinforce its role as a maladaptive regulator of post-traumatic neurodegeneration (Fig. 4E-G).

Emerging evidence challenges the traditional binary classification of microglial activation states into strictly pro-inflammatory (“M1-like”) and anti-inflammatory (“M2-like”) phenotypes. While subclusters Mic. 7 and Mic. 9 displayed multiple pro-inflammatory genetic markers-typically associated with the “M1-like” phenotype-they also exhibited concomitant upregulation of select “M2-like” signature genes. This co-expression of both pro- and anti-inflammatory markers underscores the limitations of rigid M1/M2 categorizations.

Notably, the nine identified microglial subtypes collectively demonstrated a phenotypic continuum, encompassing transcriptional profiles associated with both M1 and M2activation states, with no subtype showing exclusive allegiance to either paradigm (Fig. 4H). This spectrum-like behavior aligns with the growing consensus in TBI research that binary classifications fail to capture the pathophysiological complexity of microglial responses, particularly given the dynamic spatiotemporal heterogeneity inherent of CNS injuries.

Building upon our targeted pathway analysis of TBI-associated mechanisms, we prioritized pathways exhibiting dual regulatory capacities in neuroinflammation and cellular homeostasis. This focused investigation employed stringent bioinformatic stratification to dissect microglial transcriptional dynamics, revealing functional heterogeneity across clusters that correlated with their injury-responsive trajectories. Mic. 1 displayed a dual transcriptional identity, co-expressing homeostatic surveillance genes (*Cx3cr1*, synaptic surveillance receptor *P2ry12*, and *Tmem119*). Concurrent stress-responsive activation was evidenced by *Junb/Fos* and chaperone *Hsp90aa1*, while sustained *TGFβ* upregulation indicated microenvironmental adaptation. This transcriptional duality positions Mic. 1 at the homeostatic-injury interface, suggesting balanced transcriptional modulation of neural surveillance and inflammatory priming. Mic. 4 emerges as a chronic TBI-specific microglial subset exhibiting maladaptive homeostasis, co-retaining homeostatic surveillance genes (*Cx3cr1/P2ry12/Tmem119*) with chronic stress markers (*Junb/Fos/Hsp90aa1*) and AD-risk effectors (*Adam10/Trem2/Apoe*). This triad drives neurodegeneration through temporal functional shifts: *Trem2/Apoe*-mediated lipid dysregulation exacerbates axonal pathology, while *Adam10*-βCTF potentiates tauopathy. Synergistic *Irf1*-CD8^+^ T cell crosstalk sustains neuronal vulnerability, with chronobiological (*Crybb1/Zfhx3*) and proteostatic (*Hsp90aa1*) disruptions cementing its role as a pathological memory reservoir resistant to repopulation therapies. Mic. 5 defines an acute-phase-specific microglial state through tripartite activation: proliferative surge (*Mcm4/5/6/Hells*), NLRP3 inflammasome hyperactivity (*IL-1β/TNF*), and chemotactic amplification (*Ccl3/Ccl12/Cxcl10*). Co-expression of stress-adaptation chaperone Hsp90aa1 with proto-oncogenic Junb/Fos reveals a paradoxical proliferative-stress interface, whilst Srgn-potentiated potassium efflux escalates inflammasome activation. This dual-edged reactivity – enabling debris clearance yet risking immunopathology – underscores temporally-precise NLRP3 inhibition (e.g., MCC950) during the hyperacute window, contrasting Mic. 1’s homeostatic-injury balancing act. Mic. 6 defines an acute-phase-specific microglial state through tripartite adaptation: proliferative competence (*Mcm2/4/5/6/Hells/Stmn1*), apoptosis resistance (*Bcl2a1/isoforms/Gpx4*), and chemotactic signaling (*Ccl12/Cxcl16*). Co-retention of stress sensor *Srgn* with proliferative machinery reveals a “resilient expansion” paradigm – sustaining population dynamics under oxidative stress while countering Mic. 5’s inflammasome-driven expendability. Mic. 7 embodies acute-phase microglial adaptation via triple mechanisms: stress-proliferation (*Hsp90aa1/Fos/Junb*), apoptosis resistance (*Bcl2a1*), and NLRP3-chemokine activity (*IL-1β/TNF/Ccl3/Cxcl16*). Mic. 8 embodies subacute-phase microglial adaptation through convergent DAM signatures (*Tyrobp/Apoe-Ctsb* phagocytic-lipid axis and *Spp1/Lpl-Cst7* metabolic reprogramming), apoptosis/ferroptosis resistance (*Bcl2a1 cluster/Gpx4*), and AD-risk convergence (*Aope*-amyloidogenesis with *Hif1a*-hypoxia adaptation), Mic. 9 defines a subacute-phase microglial state through IFN-primed DAM convergence, co-activating lysosomal-phagocytic (*Tyrobp/Apoe-Ctsb*) and lipid-metabolic (*Spp1/Lpl-Cst7*) axes with apoptosis resistance (*Bcl2a1 isoforms*), hyperactive IFN signaling (*Irf7/Stat1-Parp9/14*), and AD-risk potentiation via *APOE/Trem2*-driven lipid-oxysterol dysregulation.

This single-cell sequencing analysis systematically delineates the functional heterogeneity of microglial subpopulations across both temporal (acute → subacute → chronic phases) and spatial (inflammation-metabolism-proliferation axis) dimensions following TBI. Each microglial subpopulation drives distinct pathological processes through unique transcriptional programs, highlighting the critical need for targeted interventions tailored to subpopulation-specific mechanisms. The functional plasticity of microglial subsets represents both the foundation of TBI’s pathological complexity and a promising avenue for stratified therapeutics. By integrating insights from chronobiology, metabolomics, and single-cell technologies, future therapeutic strategies have the potential to transcend conventional “one-size-fits-all” anti-inflammatory approaches. Instead, they may embrace a precision medicine framework that synergistically addresses subpopulation-specific, stage-specific, and individual-specific pathological dynamics, ultimately enabling more effective and personalized treatment paradigms (Fig. 4I,J).

## Translational models and microglia-targeted therapies

Despite the significant impact of TBI on mortality and disability, effective treatments remain challenging to identify. While many preclinical studies have demonstrated promising pharmacological interventions in animal models, none have translated into successful clinical trials^[^435-437^]^. The human brain is a complex, gyrencephalic organ composed of multiple interconnected regions, each involved in various functional networks^[^438-440^]^. Replicating the dynamic nature of human TBI in non-human models remains challenging^[^36,439,440^]^. Despite the ongoing translational challenges associated with animal models, it remains essential to rely on the critical insights gained from these models for translation to humans. Furthermore, the use of animal models continues to offer significant advantages in advancing our understanding of disease mechanisms and therapeutic development. Animal models enable controlled injury size and location, facilitating the study of specific neuropathological mechanisms and recovery. This allows for precise targeting of brain injury to isolate the role of distinct neural systems in anatomical and physiological disruption, as well as functional outcomes such as cognition, motivation, and motor control^[^441^]^. Currently, several experimental animals are utilized in different TBI models, including mice, rats, Xenopus Tadpoles, sheep, pigs, and non-human primates (NHPs). TBI is commonly studied using rodent species, including rats and mice, due to their widespread use in research models^[^25^]^. Nevertheless, rodent models exhibit notable constraints in investigating TBI-related neuropsychiatric outcomes due to their incomplete recapitulation of human pathological features. For instance, secondary axonal damage develops at an accelerated rate in rodents compared to humans and larger species such as primates. Furthermore, immature rodent models of pediatric TBI predominantly generate localized lesions rather than the widespread neuronal damage observed clinically^[^442^]^. This lack of translation is most often attributed to neuroanatomical differences between rodent and human brains. Specifically, the smaller brain, lissencephalic cerebrum, weak tentorium cerebelli, low ratio of white to gray matter^[^443^]^. Although rodents exhibit comparable sensorimotor deficits and histopathological changes to humans following TBI, they fail to develop the prolonged comatose states frequently observed clinically in human patients^[^444^]^. A critical consideration in translational research is the interspecies disparity in temporal progression of post-traumatic pathophysiological cascades. Systematic analyses of cross-species divergences across multiple TBI-related domains – including cerebral glucose metabolism, neuroinflammatory dynamics, axonal degeneration trajectories, and osmoregulatory dysfunction – demonstrate the absence of a universal temporal conversion coefficient to extrapolate rodent-derived data to human neurotrauma timelines^[^445^]^. The Xenopus tadpole model exhibits distinct advantages that synergize with mammalian systems in TBI research. Its vertebrate-conserved neurodevelopmental architecture provides unique insights into conserved injury mechanisms, while its experimental tractability has proven instrumental in investigating neurodevelopmental pathologies linked to trauma^[^445^]^. A research consortium has established a targeted impact paradigm to investigate TBI in Xenopus laevis tadpoles. This model employs precise lesions to the optic tectum – an evolutionarily conserved structure homologous to the mammalian superior colliculus and critical for visuomotor integration in amphibians^[^446^]^. Behavioral assays quantifying visual-motor coordination deficits are combined with histological analyses of neurodegenerative pathology and neuroinflammatory cascades to provide a multimodal assessment of post-TBI sequelae. The authors report that reactive microglia peak at 24 h and subsequently subside. However, they reappear at 168 h, suggesting a potential role in secondary brain injury^[^447^]^. Ovine and porcine models exhibit superior congruence with human neuroanatomical architecture compared to rodents, most notably their gyrencephalic cortical patterning and white-grey matter volumetric proportions^[^448^]^. While these anatomical homologies bolster their utility for recapitulating neurotrauma pathogenesis, interspecies divergences in biomechanical properties – such as viscoelastic tissue responses to impact forces and CSF dynamics – necessitate careful calibration of experimental parameters across models^[^449^]^. A primary advantage of ovine models lies in their neuroanatomical dimensions and structural complexity, which more closely approximate human cerebral architecture than smaller laboratory species. The adult sheep brain exhibits a cerebral mass of 130–140 g, bridging the vast neurovolumetric gap between rodents (1–2 g) and humans (1300–1400 g), with the latter being approximately two orders of magnitude larger than sheep^[^450^]^. The elevated heterozygosity rates observed in sheep populations engender demographic diversity that mirrors the genetic variability inherent to human cohorts. The inaugural ovine TBI paradigms predominantly employed FPI to replicate cerebral trauma. Contemporary approaches, however, have shifted towards CCI with surgical precision and unconstrained acceleration-impact protocols, which better approximate polytrauma scenarios observed in clinical settings^[^451-453^]^. A research group has developed a sheep mTBI model. Through histological analysis of the cortical grey matter at the impact site, they qualitatively observed neuronal structural changes that correlated with the macroscopic injury severity identified via gross examination and MRI. However, no significant astrocytic or microglial responses were detected^[^454^]^. Porcine models of DAI effectively recapitulate key neuropathological hallmarks observed in human TBI, including axonal terminal bulb formation, neurofilament aggregation and Aβ deposition, alongside phosphorylated tau species. These molecular signatures mirror the post-traumatic proteinopathy cascade implicated in chronic neurodegeneration, providing critical insights into TBI-induced secondary injury mechanisms^[^455^]^. The brain tissue of pigs subjected to rapid rotational head movements in the coronal plane to induce mild TBI was assessed. Neuropathological evaluations were conducted on specimens collected 1-year post-injury, focusing on axonal pathology, microglial morphological changes, and astrocytic reactivity. Most notably, there was an increase in microglial branching, connectivity, and the number of endpoints. These subtle changes were most evident in the periventricular WM and specific hippocampal subregions, persisting in some cases even 1-year post-injury. These ongoing morphological alterations suggest sustained changes in neuroimmune homeostasis^[^456^]^. The phylogenetic emergence of primate cortical specialization has engendered distinct multisensory-motor convergence networks across functionally segregated neocortical domains. These neuroanatomical adaptations reflect selective pressures to orchestrate hierarchical processing of environmental stimuli through cortico-subcortical feedback mechanisms, enabling ethologically relevant responses to ecological challenges^[^457,458^]^. A research consortium has utilized CCI protocols to induce focal contusions within the dorsolateral premotor cortex (M1 hand representation) of neotropical primates (*Saimiri* spp., *n* = 3). This longitudinal investigation (90-day post-injury window) employed quantitative kinematic metrics to delineate trauma-induced degradation of precision grip dynamics and object manipulation capacities, providing novel insights into cortical reorganization timelines^[^459^]^. The investigators developed a NHP model of TBI to evaluate motor function impairments and associated alterations in neural network connectivity linked to functional recovery^[^459^]^. Following TBI in NHPs, sustained microglial and macrophage activation persists in the cerebral parenchyma, accompanied by upregulation of BDNF and its cognate receptors. This expression profile implies potential neurotrophic modulation during chronic neurorestorative processes^[^98^]^. Current TBI models of various types for these experimental animals remain incomplete, with limited characterization of dynamic changes across different time points. Addressing these gaps represents a crucial focus for future research and development.

A research consortium has proposed enhancing the implementation of clinically translatable outcome measures (e.g., neuroimaging, circulatory biomarkers) in preclinical investigations, advocating for extended longitudinal protocols and the integration of big data analytics frameworks to synthesize and interpret multidimensional datasets^[^460^]^. Implementing big data analytics in preclinical studies necessitates larger cohorts than conventionally employed in animal research. Obtaining such cohorts can be achieved through multi-center dataset harmonization, which facilitates the incorporation of diverse injury phenotypes, clinical covariates, and TBI severity strata into unified analytical frameworks^[^16,461-466^]^. This review selectively presents Tables 1 and 2 summarizing preclinical and clinical intervention strategies reported to date. A critical knowledge gap persists regarding interspecies divergence in optimal dosing regimens, pharmacokinetic profiles, and target engagement fidelity for pharmacological agents within TBI research. To bolster scientific rigor and enhance translational predictability, systematic mechanistic investigations incorporating interspecies pharmacokinetic-pharmacodynamic modeling and dose-response validation are imperative for candidate therapeutics^[^16,467,468^]^.

**Table 1:** Selected preclinical interventions targeting microglia after TBI

Intervention	Animal model	Effects on microglia	Dose	Delivery system	Administration method	Outcomes	Reference
Fingolimod (FTY720)	C57BL/6 mice (CCI)	Reduced overall microglial activation while enhancing the M2/M1 ratio.	1 mg/kg	Oral	Three doses administered at 1, 24, and 48 h post-injury.	Mitigated cerebral edema, BBB disruption, and neurological deficits.	^[^469^]^
Minocycline	Eleven-day-old Rat (AHT)	Reduced microglia/macrophage reactivity in corpus callosum at 3dpi, but not 7dpi.	45 mg/kg	Intraperitoneal injection	Initial dose (45 mg/kg) immediately after the third impact, followed by 45 mg/kg every 12 h for 3 days.	No mitigation of axonal injury, neurodegeneration, or cognitive deficits, yet exacerbated memory deficits.	^[^470^]^
	C57BL/6 mice (CHI)	Reduced activated microglia accumulation	22.5 mg/kg	Intraperitoneal injection	Initial dose (45 mg/kg) at 30 min post-trauma, followed by 22.5 mg/kg every 12 h for 1 week.	Enhanced and sustained neurological recovery over 6 weeks.	^[^471^]^
Resatorvid (TAK-242)	C57BL/6 mice (CHI)	Reduced pro-inflammatory microglial activation acutely post-TBI.	3 mg/kg	Intraperitoneal injection	Administration commenced at 1 h post-TBI, following neurological severity scoring.	Prevents synaptic loss and BBB damage, promoting tissue recovery.	^[^472^]^
EPO	C57BL/6 mice (cortical cryolesion)	Suppressed microglial inflammatory responses post-injury.	5 IU/g	Intraperitoneal injection	Administration commenced post-trauma, following incision closure	Regulates microglial motility, promoting neuroinflammatory control.	^[^473^]^
	Rat (DTAI followed by 30 minutes of hypoxic ventilation)	Reduced macrophage/microglial infiltration in the corpus callosum post-intervention, acutely at 7 days with partial persistence at 14 days.	5 IU/g	Intraperitoneal injection	Administration commenced at 1 and 24 hours post-injury	Promotes sensorimotor and cognitive recovery, enhancing functional restoration.	^[^474^]^
Colony-stimulating factor 1 (CSF-1)	C57BL/6 mice (repetitive mTBI)	Modulated microglial reactivity post-injury	800 μg/kg	Intraperitoneal injection	Administration commenced at 24 h post-final impact (acute) and three times weekly for one month post-injury (chronic).	Rescues chronic cognitive dysfunction post-mTBI, with acute and delayed CSF1 administration synergistically mitigating neuropathology.	^[^475^]^
Hypothermia	Rat (LFP)	Modulated microglial autophagy-apoptosis-TLR4 axis, attenuating neuroinflammation.	Brain temperature of 32 °C	Moderate hypothermia	Cooling protocol comprised 30-minute ice immersion (induction), 4-h ice-pack maintenance (sustained), and 90-minute controlled rewarming (restoration).	Modulates microglial activation via autophagy suppression and apoptosis induction.	^[^476^]^
Hydroxychloroquine	C57BL/6 mice (CCI)	Modulated microglial activation-accumulation dynamics	50 mg/kg	Intraperitoneal injection	Administration commenced immediately post-injury, with daily dosing sustained until endpoint.	Ameliorates post-TBI neurological deficits via attenuation of neuroinflammation, alleviation of BBB disruption and cerebral edema, and enhancement of tight junction protein expression	^[^253^]^
Glycyrrhizin	Sprague-Dawley rats (FCI)	Shifted microglial activation from M1 to M2 phenotype.	10 mg/kg	Intraperitoneal injection	Administration commenced immediately post-TBI, with daily dosing sustained until endpoint.	Rescues neurological function recovery post-TBI, with administration reducing lesion volume and suppressing HMGB1 release and expression.	^[^143^]^
Rosiglitazone	C57BL/6 mice (lateral FPI)	Shifted microglial activation from M1 to M2 phenotype.	6 mg/kg	Intraperitoneal injection	Administration commenced 15 minutes prior to injury induction.	Attenuated the inflammatory response, mitigating TBI-induced axonal injury.	^[^263^]^
gp91ds-tat	C57BL/6 mice (CCI)	Shifted microglial activation from M1 to M2 phenotype.	5 mg/kg	Intraperitoneal injection	Administration commenced at 1 day post-injury, with repeated intraperitoneal administrations at 2 and 3 days post-injury.	Suppressed NOX2 expression in activated microglia at 7 days post-injury, upregulating hippocampal Arg1 and Ym1 expression, and ameliorating spatial working memory deficits.	^[^477^]^
Atorvastatin	C57BL/6 mice (CCI)	Shifted microglial activation from M1 to M2 phenotype.	1 mg/kg	Oral gavage	Administration commenced at 1 h post-TBI, with repeated administrations at 24 and 48 h post-TBI.	Suppressed neuronal apoptosis, ameliorating functional impairments.	^[^478^]^
Resveratrol	C57BL/6 mice (CCI)	Modulated microglial activation dynamics in the cerebral cortex, corpus callosum, and dentate gyrus post-mTBI, attenuating region-specific neuroinflammatory responses.	100 mg/kg	Subcutaneous	Administration commenced at 5 minutes post-mTBI, with repeated administrations at 12 hours post-mTBI.	Attenuated neuroinflammation, mitigating secondary brain injury.	^[^479^]^
Pioglitazone	C57BL/6 mice (repetitive mTBI)	Modulating microglial activation dynamics can promote pathways associated with wound healing and tissue restoration while suppressing DAM-like phenotypic shifts.	12.5 ppm/50 ppm	Oral	Incorporated into standard soy protein-free rodent chow (12.5 ppm and 50 ppm diet), administered ad libitum for 3 months.	Attenuated spatial learning and memory impairments at 6 months post-injury in the cortex, hippocampus, and corpus callosum, suppressing reactive microglial and astroglial marker expression, mediated via PPARγ activation.	^[^480^]^
Curcumin	C57BL/6 mice (CCI)	Shifted microglial polarization towards an M2 phenotype, suppressing C1ql3 expression.	200 mg/kg	Intraperitoneal injection	Administration commenced 15 minutes post-TBI induction.	Alleviated TBI-induced cerebral injury, attenuating IL-1β and IL-6 levels.	^[^481^]^
	C57BL/6 mice (Feeney weight-drop contusion)	Attenuated TLR4-positive microglial activation, suppressing MyD88/NF-κB pathway engagement and pro-inflammatory signaling.	100 mg/kg	Intraperitoneal injection	Administration commenced 15 minutes post-TBI induction.	Ameliorated post-TBI clinical outcomes, attenuating acute microglial/macrophage activation and neuronal apoptosis.	^[^242^]^
SMM-189	C57BL/6 mice (focal left-side cranial blast)	Shifted microglial activation from M1 to M2 phenotype.	6 mg/kg	Intraperitoneal injection	Administration commenced 2 h post-blast and continued for 14 days in the neuronal loss study; in the pCREB study, administration was limited to 3 days.	Rescued damaged neurons and alleviated functional deficits resulting from TBI, attenuating neuronal apoptosis.	^[^482^]^
	C57BL/6 mice (focal blast model of closed-head mild TBI)	Shifted microglial activation from M1 to M2 phenotype.	6 mg/kg	Intraperitoneal injection	Administration commenced 2 h post-blast and continued for 14 days.	Alleviated motor, visual, and emotional deficits, mitigating neurological dysfunction.	^[^483^]^
CSF1R inhibitor (PLX5622)	C57BL/6 mice (CCI)	Targeted depletion of hypertrophic microglia enabled repopulation of ramified cells, suppressing NOX2/NLRP3-driven neuroinflammation and oxidative stress	1200 ppm	Oral	Incorporated into standard AIN-76A rodent chow (1200 ppm diet) by Research Diets, administered ad libitum for 7 days (resulting in 95% microglial depletion).	Attenuated cortical and hippocampal neurodegeneration, promoting sustained motor and cognitive recovery post-TBI, mitigating post-TBI neurological sequelae.	^[^484^]^

**Table 2:** Selected clinical interventions targeting microglia after TBI

Drug	Diagnosis	Study design and number of patients	Outcomes	Safety	Development Phase	Reference
Minocycline	Moderate-severe TBI, GCS score 3–12	Following an 800 mg loading dose administered to all participants, maintenance doses of 200 mg twice daily (BID) for Tier 1 (*n* = 7) and 400 mg BID for Tier 2 (*n* = 8) were administered orally for 7 consecutive days.	The higher-dose Tier 2 group demonstrated a trend towards greater reduction in Disability Rating Scale (DRS) scores compared to Tier 1 at 12 weeks.	Transient elevation in liver function tests (resolved within 1 week)	Phase I	^[^485^]^
				No infections reported in either group.		
	Moderate-severe TBI	A single-center observational cohort study enrolled 15 patients with moderate-to-severe TBI (≥6 months post-injury, focal lesions, no neurosurgery), allocated to either minocycline 100 mg orally twice daily or no drug for 12 weeks.	Primary: Reduced microglial activation	The treatment was generally well-tolerated, with two mild adverse events reported: transient nausea and vomiting (resolved by reducing the dose to 100 mg once daily) and unilateral hearing impairment (resolved spontaneously following decongestant administration).	Phase II	^[^486^]^
			Secondary: Increased plasma neurofilament light			
Recombinant erythropoietin (EPO)	Moderate-severe TBI, GCS score 3–12	Multicenter RCT with pre-planned long-term follow-up (median 6 years post-injury). Original trial: 603 patients; Follow-up cohort: 356 survivors.	Primary: No long-term survival benefit. Secondary: Sliding-scale GOSE improvement.	No significant safety concerns	Phase III	^[^487^]^
Prophylactic hypothermia (33°C-35°C)	Severe TBI, GCS score 4–7	The POLAR-RCT multicenter trial in 6 countries enrolled 511 patients randomized to prophylactic hypothermia (*n* = 266) or normothermia (*n* = 245); hypothermia (33–35°C) was initiated at a median of 1.8 h post-TBI, maintained ≥72 h, followed by gradual rewarming (median 22.5 h), while normothermia (37°C) was maintained with cooling wraps. Primary outcomes were assessed in 466 patients at 6 months.	Primary: No difference in favorable neurological outcomes at 6 months (48.8% vs 49.1%, *P* = 0.94). Secondary: No improvement in GOS-E ordinal scores (*P* = 0.88) or mortality rates (21.1% vs 18.4%, *P* = 0.45).	No significant safety concerns	Phase III	^[^488^]^
Atorvastatin	Severe TBI, GCS score 3	A 29-year-old male with severe TBI (GCS 3/15) received neurosurgical care, 2-year rehabilitation, and longitudinal imaging/functional assessments over 4.5 years post-injury, including oral atorvastatin (20 mg/day) during hydrocephalus monitoring.	Reduced third ventricle span (21%↓) and Evan’s index (16%↓); improved motor (17→56/100) and cognitive (15→23/30) scores.	No significant safety concerns	Early exploratory clinical research (single-patient case study).	^[^489^]^
Recombinant human interleukin-1 receptor antagonist	Severe diffuse TBI	Randomized controlled trial (RCT) enrolled 20 patients with severe diffuse TBI (within 24 h post-injury), randomized to receive rhIL1ra 100 mg once daily for 5 days or no drug (control group).	The intervention altered brain cytokine profiles, elevating M1 markers (GM-CSF, IL1) and MCP-1, while controls showed upregulated M2 markers (IL4, IL10, MDC), with effect size dependent on intracerebral drug concentration.	No significant safety concerns	Early-phase clinical research	^[^490^]^

Detailed studies are referenced for additional information. CCI: controlled cortical impact; AHT: abusive head trauma; CHI: closed head injury; DTAI: diffuse traumatic axonal injury followed; mTBI: mild traumatic brain injury; LFP: Lateral Fluid Percussion; FCI: focal contusion injury; Lateral FPI: lateral fluid percussion injury.

## Conclusions and future prospect

TBI triggers a dynamic neuroinflammatory response, with microglia playing a central role in influencing both pathological progression and tissue restoration. While significant advances have been made in elucidating the molecular and cellular mechanisms underlying microglial activation, many critical questions remain unresolved. The conventional M1/M2 polarization framework is increasingly recognized as an oversimplification, failing to capture the dynamic and heterogeneous nature of microglial phenotypes across different stages of injury. Emerging single-cell transcriptomic analyses have provided unprecedented insights into microglial subpopulations, revealing distinct temporal and functional transitions that may serve as novel therapeutic targets.

A major challenge in the field lies in translating preclinical findings into effective clinical interventions. Existing animal models, though valuable, exhibit inherent limitations in replicating the complexity of human TBI, necessitating refined modeling approaches that better mimic lesion heterogeneity and disease progression. Additionally, current therapeutic strategies for microglial modulation remain largely exploratory, with limited success in clinical trials. Achieving therapeutic efficacy requires a shift from broad-spectrum immunosuppression towards precision-targeted neuroimmune interventions that account for injury phase-specific microglial functions.

Future research should prioritize several key directions. First, integrating advanced omics technologies, including spatial transcriptomics and proteomics, will enhance our understanding of microglial heterogeneity and functional plasticity over time. Second, the development of human-relevant TBI models, such as organoid-based or chimeric approaches, may help bridge the translational gap between experimental and clinical research. Third, investigating the crosstalk between microglia and other CNS-resident and peripheral immune cells will be critical for identifying synergistic therapeutic targets. Finally, translating microglia-based interventions into the clinic will require rigorous validation through multicenter collaborative studies, biomarker-driven patient stratification, and personalized treatment strategies.

By addressing these challenges, the field can move towards a more comprehensive understanding of microglial dynamics in TBI and leverage this knowledge to develop novel, phase-specific neuroimmune therapies. With continued progress, precision-targeted microglial modulation holds the potential to improve neurological outcomes and mitigate the long-term consequences of TBI.

## Data Availability

The data in this review is not sensitive in nature and is accessible in the public domain. The data is therefore available and not of a confidential nature.
